# Role of imaging in visceral vascular emergencies

**DOI:** 10.1186/s13244-020-00913-3

**Published:** 2020-10-15

**Authors:** Ali Devrim Karaosmanoglu, Aycan Uysal, Deniz Akata, Mustafa Nasuh Ozmen, Musturay Karcaaltincaba

**Affiliations:** 1 Department of Radiology, Hacettepe University School of Medicine, 06100 Ankara, Turkey; 2 Department of Radiology, Gulhane Training and Research Hospital, 06010 Ankara, Turkey

**Keywords:** Acute abdomen, Visceral vessels, Vascular emergency, Aneurysm, Thrombosis, Dissection

## Abstract

Differential diagnosis in non-traumatic acute abdomen is broad and unrelated diseases may simulate each other from a clinical perspective. Despite the fact that they are not as common, acute abdominal pain due to diseases related to visceral vessels may be life-threating if not detected and treated promptly. Thrombosis, dissection, and aneurysm in the abdominal visceral arteries or thrombosis in visceral veins may cause acute abdominal pain. Imaging with appropriate protocoling plays a fundamental role in both early diagnosis and determination of the treatment approach in these cases where early treatment can be life-saving. Computed tomography (CT) appears to be the most effective modality for the diagnosis as it provides high detail images in a very short time. Patient cooperation is also a less concern as compared to magnetic resonance imaging (MRI). As the imaging findings may sometimes be really subtle, diagnosis may be difficult especially to inexperienced imagers. Correct protocoling is also very critical to detect arterial abnormalities as visceral arterial abnormalities may not be detectable in portal phase only abdominal CT scans. In this article, we aimed to increase awareness among imaging specialists to these not very common causes of acute abdomen.

## Key points

- Abdominal visceral vascular disorders may present with acute abdominal pain and may have life-threatening consequences. Therefore, prompt evaluation of these patients is of fundamental importance.
- Appropriate protocoling of the imaging studies and familiarity to the radiologic findings of several different visceral vascular abnormalities may facilitate the diagnosis and may allow better treatment planning.
- Imaging findings may be subtle in these patients and early diagnosis may be of crucial importance in preventing conditions of high morbidity and mortality.

## Introduction

Acute abdominal pain (AAP) is a common complaint among patients presenting to the emergency department (ED). AAP is approximately 5% of all ED visits in the USA in 2017 [1]. Obtaining a detailed and focused medical history is the key to correct diagnosis; however, imaging still plays a critical role. The differential diagnosis for AAP is broad, ranging from life-threatening conditions to benign self-limiting diseases. Acute appendicitis, acute diverticulitis, acute cholecystitis, and bowel obstruction are among the most frequently encountered causes for AAP. In addition to these common conditions, pathology of the visceral abdominal vessels should also be considered in the differential diagnosis [2]. Imaging findings related to abdominal vessels may be subtle; therefore, imaging specialists should be familiar with these conditions in order to prevent high morbidity and mortality. Computed tomography (CT), ultrasonography (US), and magnetic resonance imaging (MRI) may all be used in diagnosis with CT being the most frequently utilized modality.

The aim of this manuscript is to systematically review and present the imaging features of various visceral vascular emergencies to facilitate diagnosis in order to guide appropriate treatment.

## Hepatic vascular system

The liver is a very vascular organ with its unique vascular anatomy. Portal vein (PV) and hepatic artery (HA) provide the main inflow, whereas hepatic veins (HV) represent the main outflow tract. The disruption in any of these complex vascular systems may cause potential catastrophic outcomes.

### Portal vein

PV is the main supplier of the liver parenchyma. It is formed with the union of the splenic vein (SV) and the superior mesenteric vein (SMV). PV functions as a filter for the blood flow from the digestive system, spleen, pancreas, and gallbladder. Several conditions affecting the flow in the portal vein may cause severe abdominal pain which may prompt ED visits.

### Acute portal vein thrombosis

Acute portal vein thrombosis (or pylethrombosis) (PVT) is an uncommon condition that may present with AAP, especially when the SMV is also involved [3]. Enlarged spleen and ascites may be seen in 30% of the cases [4]. Both bland and tumor thrombi may cause acute PV obstruction and imaging plays a key role both for diagnosis and differential diagnosis. Clinical symptoms may be subtle but severe abdominal pain, vomiting, and diarrhea may also be seen as presenting symptoms. Acute decompensation in known cirrhotic patients may also be observed as the main complaint [5]. Early diagnosis is critical for therapeutic intervention. Portal hypertension and liver failure may develop in these patients without adequate treatment.

### Bland thrombus

**Fig. 1:**
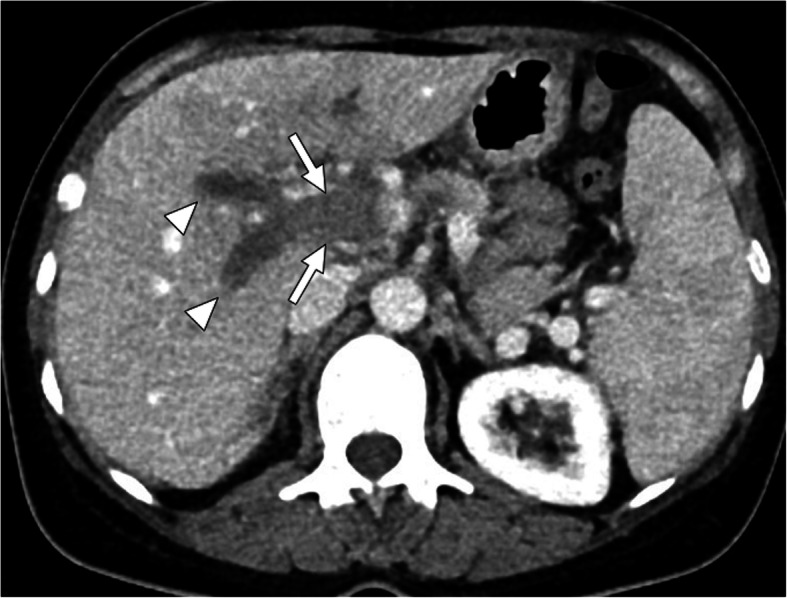
Bland thrombus. A 49-year-old female with known antiphospholipid antibody syndrome (APS) now presenting with severe right upper quadrant pain and elevated liver function tests. Axial post-contrast abdominal CT image demonstrates diffuse acute thrombosis of the main portal vein (arrows) and its intrahepatic branches (arrowheads)

**Fig. 2:**
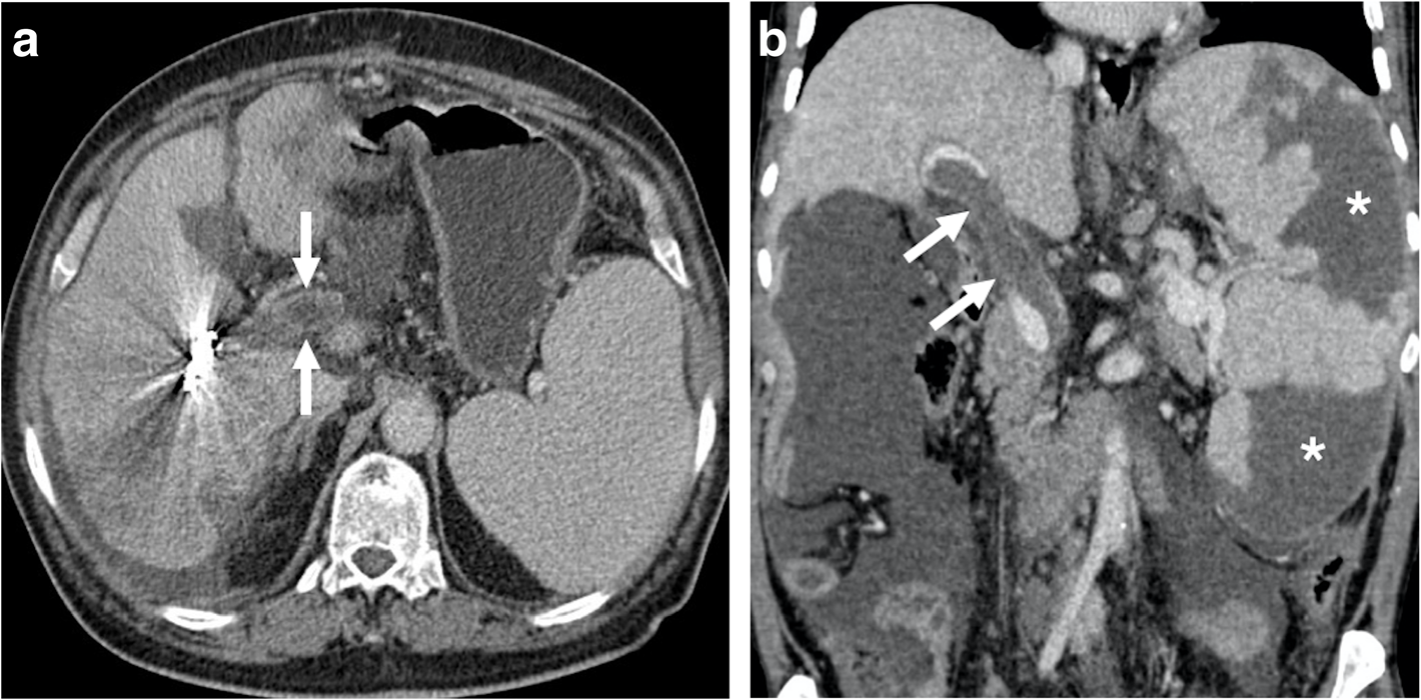
Two patients with iatrogenic portal vein thrombosis. **a** A 65-year-old male with known HCC underwent selective right portal vein embolization for surgical resection. The patient presented with severe epigastric and right upper quadrant pain 14 days after the procedure. Axial post-contrast abdominal CT image demonstrated acute main portal vein thrombosis (arrows). Also, note metallic artifacts in the right liver lobe secondary to embolization coils. **b** A 55-year-old male with known Hodgkin lymphoma underwent splenic artery embolization because of pancytopenia induced by hypersplenism presents with persistent right upper quadrant pain and elevated liver enzymes. Coronally reformatted post-contrast abdominal CT image demonstrated diffuse portal vein thrombosis (arrows). Also, note is made of diffuse splenomegaly and extensive parenchymal infarcts (asterisks)

Hematologic disorders that favor procoagulant states, intraabdominal inflammation, and cirrhosis are the most common underlying reasons for acute bland PVT [6, 7] (Fig. 1). Iatrogenic bland PVT may also be observed after percutaneous angiographic interventions (Fig. 2). US and CT are the main imaging tools for early diagnosis of PVT. On gray-scale US, heterogeneously hypoechoic thrombus within the vein lumen is the main finding. The complete absence of flow signal on color Doppler US is the other main ancillary finding.

CT is the main modality for acute assessment of PVT. The associating complications, such as enteric ischemia, intraabdominal collections, and associating malignancies may also be detected with high accuracy. The extent of the thrombus may also be fully assessed with CT. The lack of enhancement within the vein lumen and enlarged portal vein are the two main classic imaging features of bland thrombus. The absence of any contrast enhancement within the thrombus is one of the most helpful findings for differentiating bland PVT from its malignant counterpart. Parenchymal perfusion abnormalities, with increased liver enhancement on arterial phase and associated decreased parenchymal enhancement in the venous phase, are also observed. Acute bland thrombus is strictly limited to the vein lumen and does not extend outside the confines of the vessel wall, which is another important feature against tumor thrombus [8].

### Pylephlebitis

Pylephlebitis is a rare clinical condition characterized by suppurative thrombosis of the portal vein. Pylephlebitis is a serious medical condition with high mortality [9, 10]. As the delay in diagnosis may have grave consequences, early imaging and correct diagnosis are critical [9]. The process typically starts from mesenteric veins with subsequent extension into the PV. The most common underlying clinical condition is diverticulitis; however, urinary infections, acute appendicitis, pelvic infections, and biliary diseases have all been implicated in the etiology [11].

**Fig. 3:**
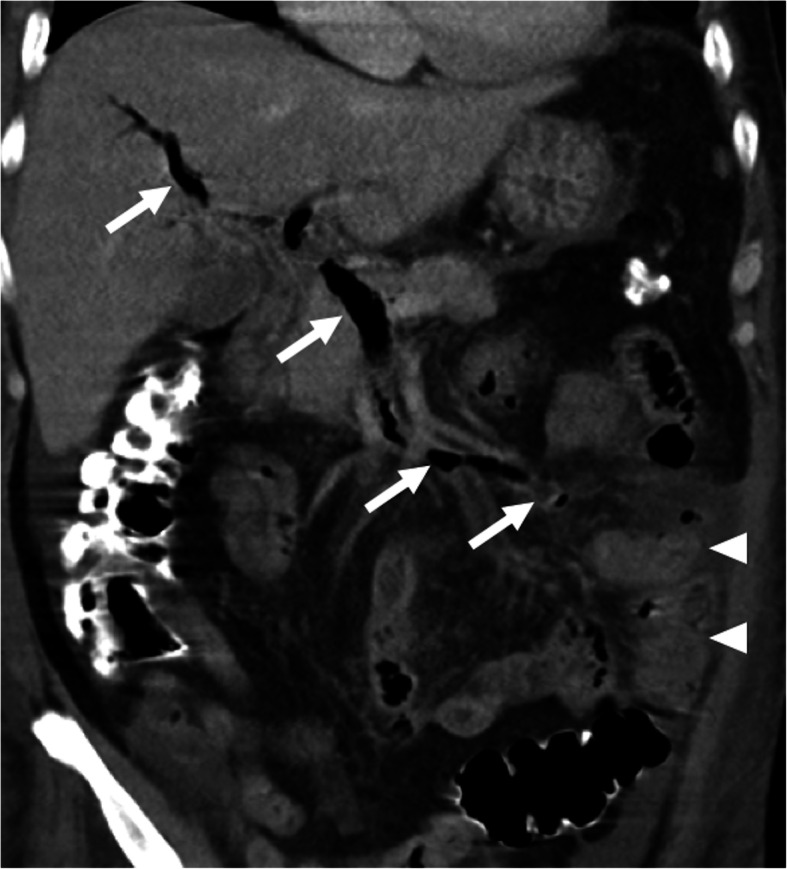
Pylephlebitis. A 56-year-old man with known diabetes presented with acute onset abdominal pain and fever. Coronally reformatted post-contrast abdominal CT image demonstrated diffuse gas and thrombosis within the porto-mesenteric venous axis (arrows). Also, note is made of diffuse wall thickening (arrowheads) in the distal jejunal segment and inflammatory changes within the adjacent mesentery. Laparoscopic resection and histopathological analysis revealed acute jejunal diverticulitis

US, CT, and MRI may all be used in the diagnosis but among all these modalities CT appears to be the most commonly utilized. With CT, whole abdominal solid organs and vessels may be scanned in a very short period of time with an excellent accuracy. The ability of creating multiplanar reformatted images with this modality also offers another advantage for both detecting and assessing the extension of the thrombus into the portal vein. With CT, the underlying intraabdominal conditions may also be detected with high accuracy. The detection of air within the thrombus may also better delineated with CT as a hypodense focus within the thrombosed PV (Fig. 3). In addition to main portal vein branches, side branches may also be involved in an isolated fashion with intact main portal vein trunk.

### Tumor thrombus

Hepatocellular carcinoma (HCC) is by far the most common cause of tumoral PVT. As the presence of tumoral thrombus gravely affects the patient outcome, early diagnosis and intervention is critical [12]. Tumoral invasion by HCC may be the first clinical presentation of HCC [13] and patients may present to ED as the first sign of the disease. The clinical findings are generally nonspecific but right upper quadrant pain and elevated liver enzymes may suggest portal vein tumor thrombus in relevant clinical context. CEUS may be used for the detection and characterization of PVT as it provides differential diagnosis between malignant and benign PVT in certain patients [14, 15].

**Fig. 4:**
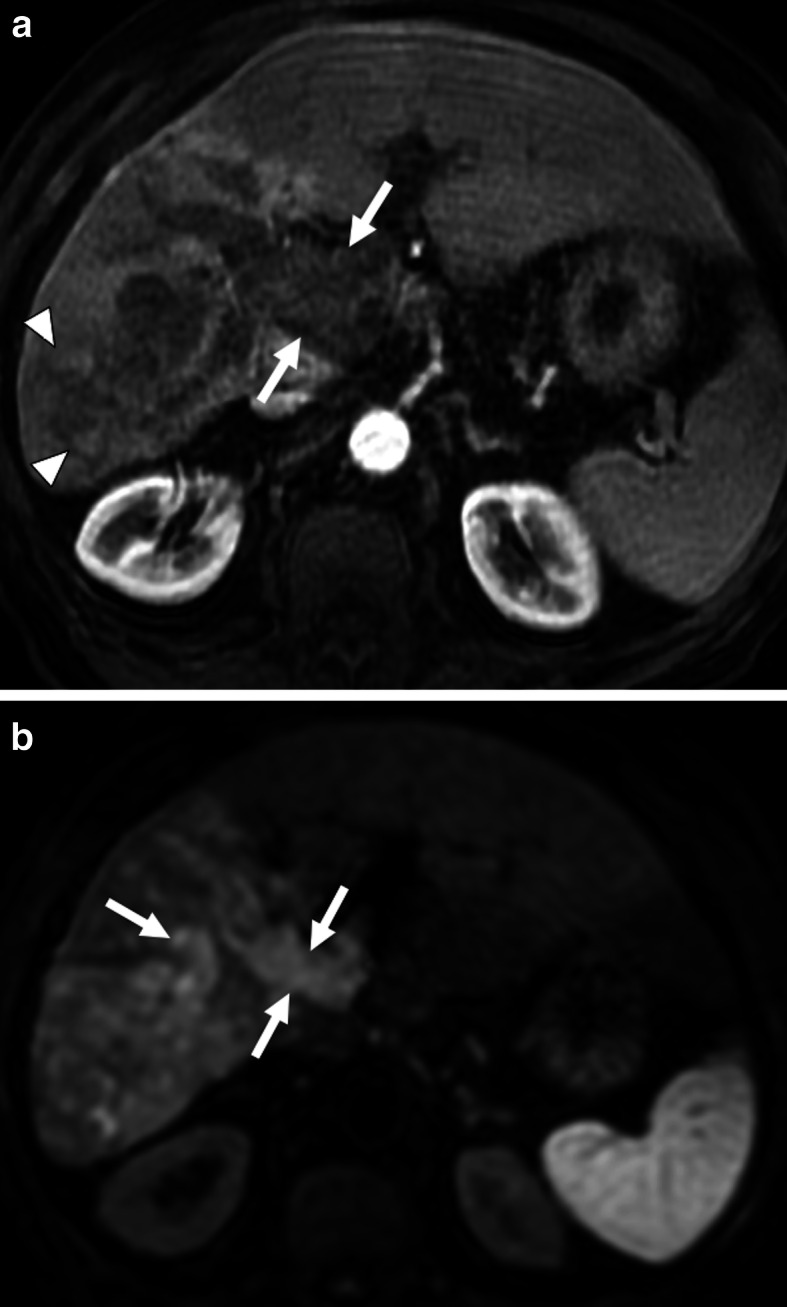
Tumor thrombus. A 76-year-old male with known cirrhosis presented with right upper quadrant pain and elevated liver enzymes. **a** Axial post-contrast arterial phase T1W MR image demonstrates HCC focus within the right liver lobe (arrowheads) with associating tumor within the main and right portal vein branches (arrows). Note contrast enhancement of the thrombus with extending outside the confines of the main portal vein. **b** DWI image shows high signal intensity representing diffusion restriction of the thrombus within the main portal vein (arrows)

The detection of enhancement within the thrombus by contrast-enhanced CT and MRI studies and extension of the thrombus outside the confines of the vessel wall are the two most important indicators favoring a malignant process over bland thrombus. On MRI, the presence of vessel expansion, continuity of the PVT with the tumor, high T2 signal intensity, and diffusion restriction are other imaging features of tumoral thrombus that may be helpful for differential diagnosis [16] (Fig. 4). Also, 3D contrast-enhanced MRA may be very useful for this purpose.

In addition to HCC, several other neoplastic processes may, not infrequently, cause tumoral PVT, such as liver metastases, gallbladder tumors, and cholangiocarcinoma [17–19]. Tumor thrombus patients may also present with acute symptoms of hepatic venous outflow obstruction

### Mesenteric venous thrombosis

Mesenteric venous thrombosis (MVT) is an uncommon but potentially life-threatening clinical condition accounting for 5–15% of all mesenteric ischemic events. As is seen in mesenteric arterial thrombosis, the most serious clinical complication is bowel ischemia and subsequent infarction [20].

Hypercoagulable states or prothrombotic disorders, myeloproliferative neoplasms, cancer (most frequently originating from the pancreas and liver), inflammatory conditions (acute pancreatitis, inflammatory bowel disease, and diverticulitis), recent surgery, portal hypertension, and miscellaneous causes such as oral contraceptives or pregnancy may be counted among the predisposing factors. Clinical symptoms are mostly nonspecific and diagnosis may, therefore, be delayed [21, 22].

**Fig. 5:**
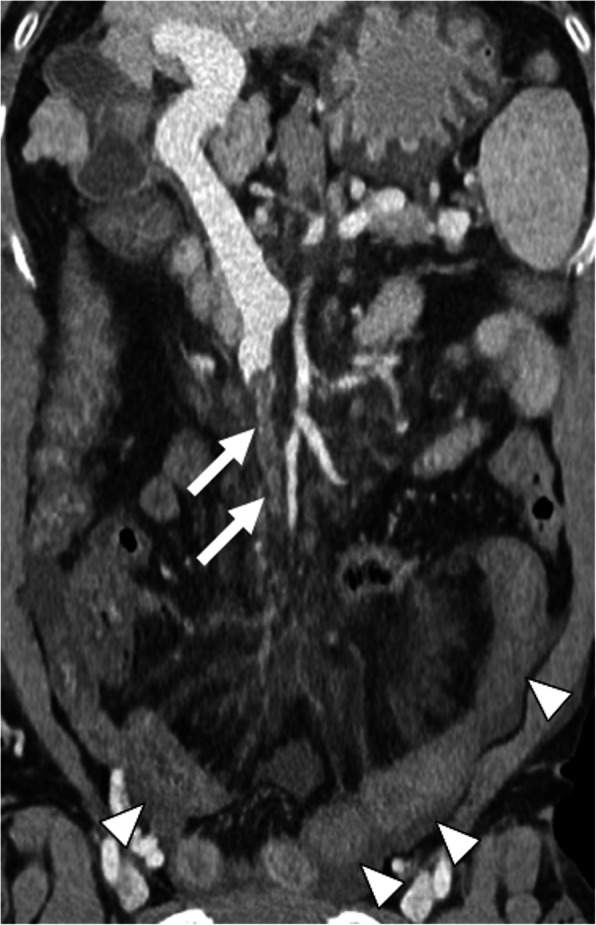
SMV thrombosis. A 57-year-old female with known history of hepatitis B-related cirrhosis and Behcet’s disease presented to ED with severe abdominal pain and melena. Post-contrast curved MPR CT image shows thrombosed SMV (arrows) with associating severe mesenteric edema. Also, note is made of thickening of small bowel and colon segments (arrowheads) suggestive for venous bowel ischemia. Emergent surgery confirmed imaging findings and the patient underwent extensive bowel resection. The patient expired 3 days after the surgery

**Fig. 6:**
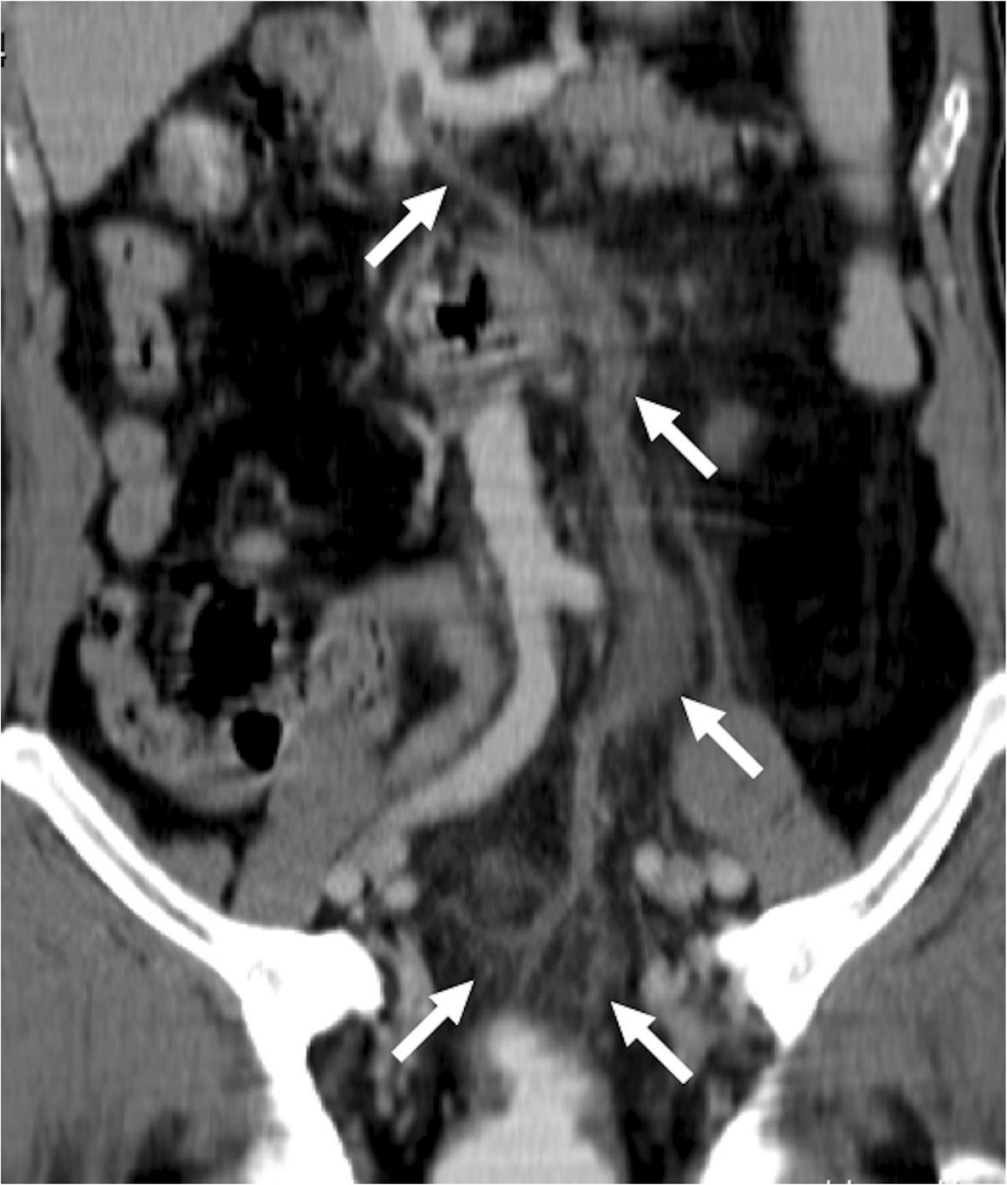
IMV thrombosis. A 70-year-old man presented to ED with severe melena, fever, and abdominal pain. Coronally reformatted post-contrast CT image showed thrombosed IMV (arrows) due to sigmoid colon diverticulitis

Contrast-enhanced CT is the preferred modality of choice in patients with suggestive symptoms for acute MVT. With CT, mesenteric veins may be directly visualized with other imaging features concerning for mesenteric ischemia or infarction (Fig. 5). Endoluminal thrombus is typically seen as a luminal filling defect on post-contrast images (Fig. 6). The detection of air within the mesenteric veins and bowel wall is typically seen in advanced stage. Mesenteric edema, bowel wall thickening and vascular engorgement may also be detected [20, 21].

### Hepatic veins

#### Budd-Chiari syndrome

Hepatic veins are the main venous outflow tract of the liver. Acute Budd-Chiari syndrome (BCS) represents the acute thrombosis of the hepatic veins and subsequent venous engorgement of the liver. Venous outflow obstruction leads to parenchymal congestion and increased sinusoidal pressure. This venous stasis leads to altered portal flow and these two factors lead to ischemic injury and subsequent parenchymal damage [23]. BCS is a rare vascular disorder of the liver with an estimated incidence of 0.1–0.8/1,000,000 per year and a prevalence of 1.4–2.4/1,000,000 per year in the USA [24]. The clinical symptoms may range from subtle symptoms to fulminant liver failure [23]. Hematologic disorders and thrombotic diathesis account for 75% of all cases and several predisposing factors may also be detected in a single patient [23, 25]. Early diagnosis is critical for planning treatment and preventing complications.

Doppler US is typically the first imaging modality to be used in acute BCS. It can effectively detect the thrombosed hepatic veins but early depiction of venous thrombus may be difficult in patients with difficult body habitus.

**Fig. 7:**
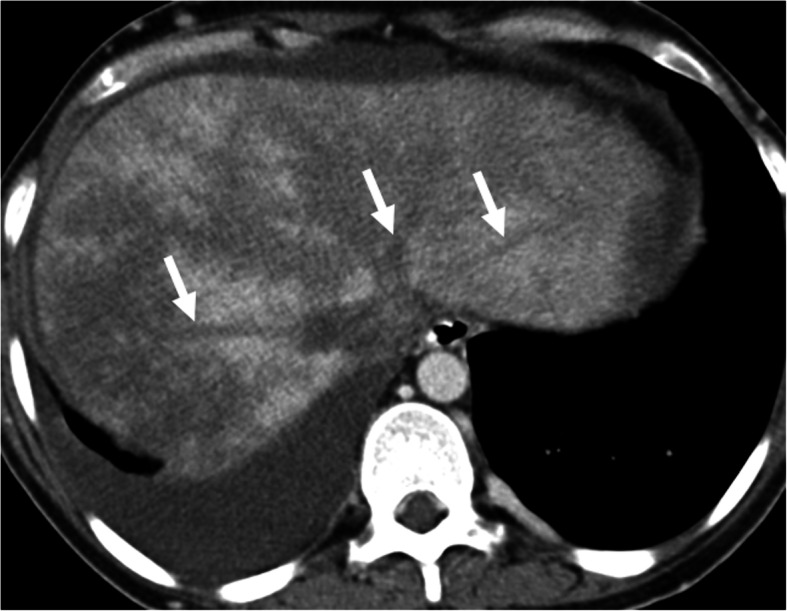
Budd-Chiari syndrome. A 29-year-old female with known Behcet’s disease presented with acute and severe right upper quadrant pain and abdominal distension. Abdominal US study showed fresh thrombus within the hepatic veins (not shown). Subsequent venous phase axial CT scan shows thrombosed hepatic veins (arrows). The liver parenchyma demonstrated heterogeneous contrast enhancement with severe parenchymal heterogeneity

**Fig. 8:**
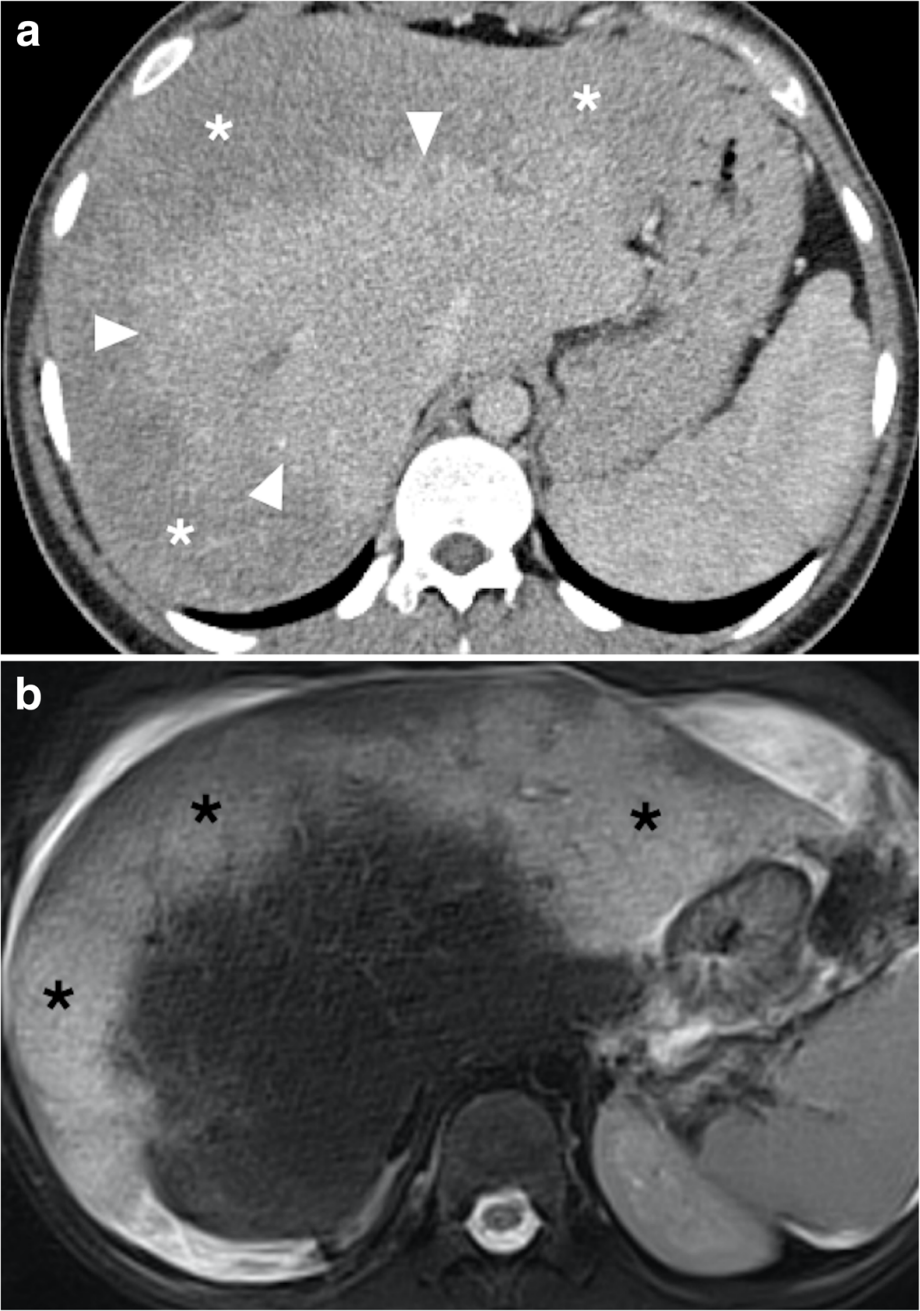
Budd-Chiari syndrome. A 23-year-old male with factor 5 Leiden mutation presented with acute epigastric pain and elevated liver enzymes. Abdominal US study demonstrated fresh thrombus in the hepatic veins and hepatomegaly. **a** Axial plane post-contrast CT image showed reduced perfusion within the periphery of the hepatic parenchyma (asterisks) with relatively normal enhancing central parenchyma (arrowheads). **b** Axial T2W MR image shows diffuse edema and congestion within the peripheral portion of the liver parenchyma (asterisks) which corresponds to non-enhancing liver parenchyma on CT

CT clearly shows the obstructed hepatic veins and associating parenchymal changes (Fig. 7). Peripheral parenchymal areas are typically more edematous and congested than the central liver and demonstrate decreased contrast enhancement as compared to stronger enhancement of the central parts of the liver parenchyma (Fig. 8). The peripheral liver parenchyma usually appears hypodense on contrast-enhanced CT and hypointense on contrast-enhanced MRI in the arterial phase. This finding is related to elevated post-sinusoidal pressure. Caudate lobe may demonstrate better venous enhancement due to its unique venous drainage pattern. A “flip-flop” pattern may occur in the portal venous phase as the central part of the liver demonstrates low attenuation due to contrast wash-out and gradual increase of the peripheral attenuation [26].

The liver appears diffusely hypodense on CT and of low signal intensity on T1-weighted MR images. T2-weighted sequences usually show heterogeneously increased signal intensity in the peripheral liver parenchyma. Ascites may also be detected.

The chronic parenchymal congestion with the formation of focal parenchymal nodules may eventually cause a classical morphological situation also called as the “nutmeg liver.”

### Hepatic artery

#### Hepatic artery aneurysm and pseudoaneurysms

True hepatic artery aneurysms (HAA) are rare and account for 20% of all visceral artery aneurysms [27]. The etiology is mostly due to degenerative and dysplastic processes with atherosclerosis being the most commonly encountered factor [28]. The risk of rupture is highly variable, ranging from 20 to 80% [27]. Both HAAs and pseudo-HAAs follow the contrast enhancement pattern of the originating artery. Both arterial and portal venous phase images should be acquired as some pseudoaneurysms with a narrow neck may enhance only in the venous phase and may be easily missed on the arterial phase images [29].

**Fig. 9:**
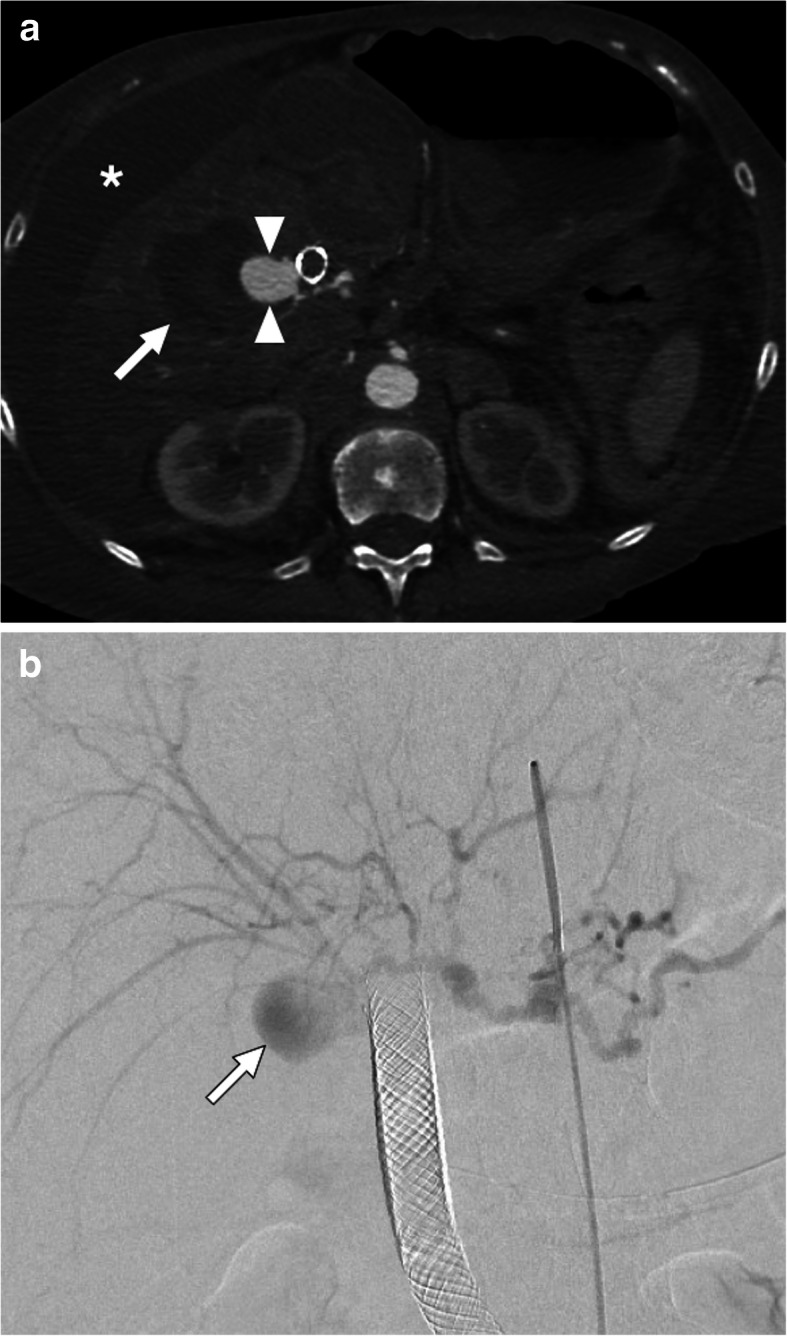
Hepatic artery pseudoaneurysm. A 69-year-old female with known inoperable obstructive hilar cholangiocarcinoma cancer treated with palliative biliary stenting. About 3 h after the procedure, the patient experienced severe right upper quadrant pain, hematemesis and hypotension. **a** Axial post-contrast arterial phase CT image demonstrates a large pseudoaneurysm (arrowheads) right next to the metallic biliary stent. Also, note is made of newly developed intraparenchymal hematoma (arrow) and intraperitoneal high-density free fluid suggestive of hemoperitoneum (asterisk). **b** Emergent catheter angiography confirmed the presence of pseudoaneurysm (arrow) originating from the right hepatic artery. The pseudoaneurysm was subsequently embolized with coils and the patient recovered rapidly after the procedure

**Fig. 10:**
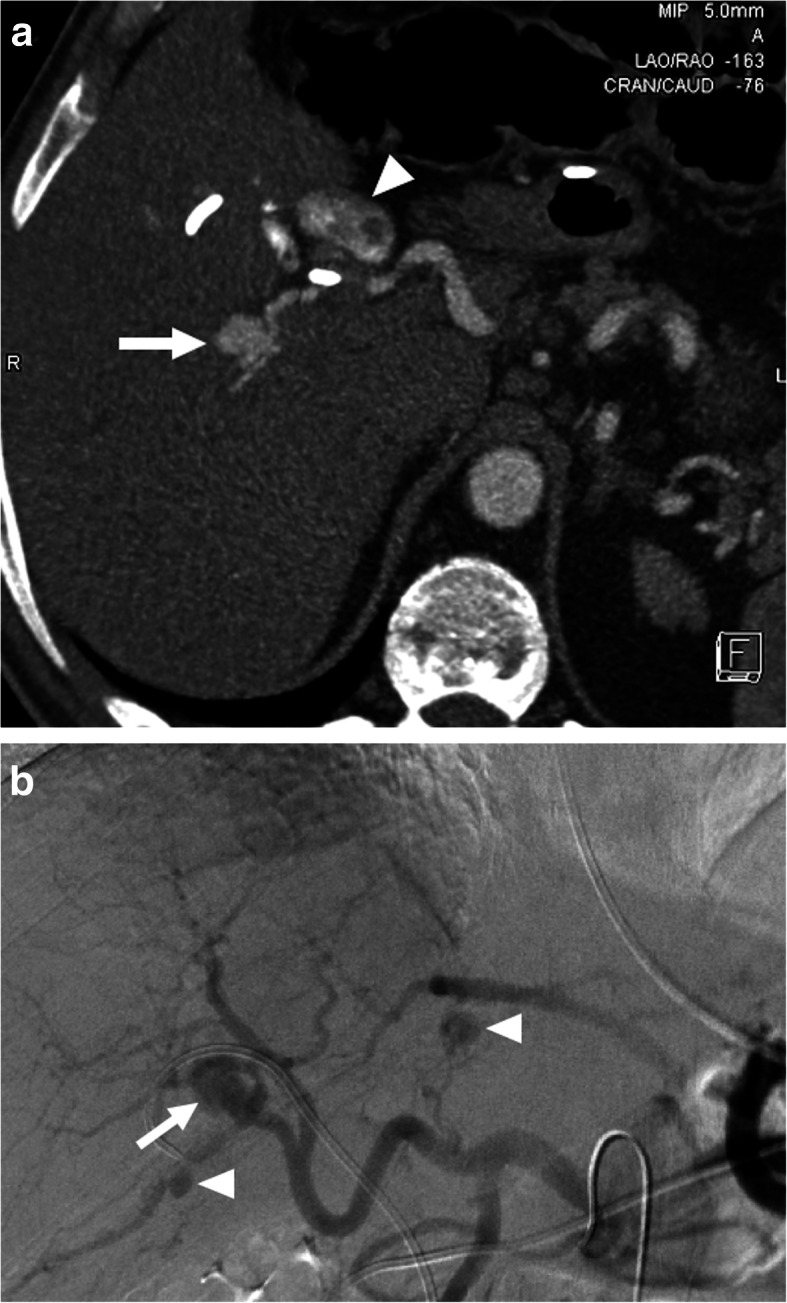
Hepatic artery pseudoaneurysm. A 42-year-old male with no known significant past medical history presented to emergency department and (ED) with severe right upper quadrant pain and hematemesis. Emergent upper gastrointestinal tract endoscopy demonstrated active fresh blood extravasation through the ampulla of Vater. A plastic stent was placed into the common hepatic duct. **a** Axial plane post-contrast arterial phase CT image showed multiple pseudoaneurysms within the liver parenchyma. The largest was seen to be originating from the right hepatic artery (arrow). Also noted was contrast within the gallbladder lumen (arrowhead) suggestive for hemobilia. **b** The largest aneurysm was confirmed with catheter angiography (arrow). Smaller pseudoaneurysms were also noted in different segments of the liver (arrowheads)

Pseudo-HAAs usually occur due to iatrogenic reasons (biopsy, biliary interventions, liver transplant, biliary interventions, etc.) or trauma and they also have a high risk for rupture and severe bleeding [30] (Fig. 9). Rheumatologic diseases may also rarely cause hepatic artery pseudoaneurysms (Fig. 10). Pseudo-HAAs are usually seen as enhancing intrahepatic masses. However, they may also be detected extrahepatically when they originate from the common or proper hepatic arteries. Pseudoaneurysms have a very fragile wall which may be prone to rupture. Their contours are mostly irregular and they typically have a saccular shape. Hematomas surrounding the pseudoaneurysms are frequently observed at the time of diagnosis [29, 31]. As imaging findings may be extremely subtle or nonexistent in the acute phase, follow-up imaging may be necessary in patients where clinical suspicion is high for HA pseudoaneurysm [32].

## Renal vasculature

Kidneys are highly vascular organs and receive 20% of the cardiac output via bilateral renal arteries [33]. US and CT are the two most commonly used imaging modalities for assessing the renal vasculature. Protocoling is essential for detecting vascular abnormalities and CT provides high temporal and spatial resolution with less demand for patient compliance as compared to US. Several emergent situations affecting the renal vasculature may be quickly assessed with CT.

### Renal artery embolism

Embolic disease of the renal arteries is a rare clinical situation [34]. The heart appears to be the most common source of embolism with atrial fibrillation, valvular diseases, and myocardial infarction among the common predisposing situations [35]. Renal arteries are the least commonly affected arterial structure with arterial thrombosis. In a series of 621 patients with peripheral arterial embolism, the renal arteries were affected in only 2% of these patients [36]. Acute flank pain with associated hematuria, nausea, vomiting, and hypertension are the common presenting symptoms. Serum lactate dehydrogenase elevation in the serum is reported to be the most sensitive biomarker for renal infarction [37].

**Fig. 11:**
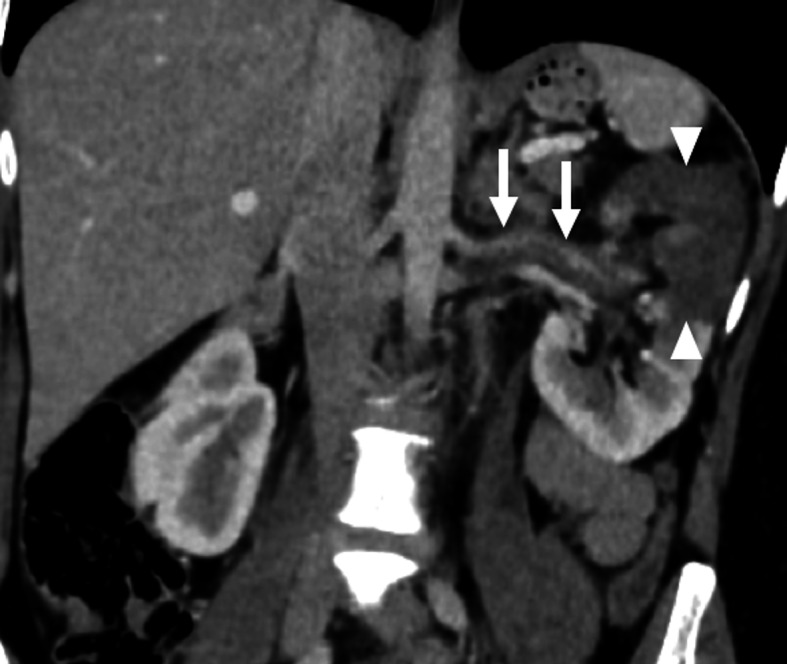
Renal artery embolism. A 47-year-old female with a history of atrial fibrillation presented with acute left flank pain and massive hematuria. Coronally reformatted post-contrast abdominal CT image demonstrates endoluminal embolus within the left renal artery (arrows). There was also a large parenchymal infarct area (arrowheads) in the upper pole of the left kidney

CT plays an important role in diagnosing this rare acute clinical situation. Proper timing is important as renal arteries are much better appreciated on arterial phase images. Coronal and sagittally reformatted images may be extremely helpful, in addition axial plane images, for detecting the endoluminal filling defects (Fig. 11). Venous and nephrogram phase images should also be included in clinically suspected cases to detect associated renal infarcts. The typical appearance of renal infarction on post-contrast CT is single or multiple foci of non-enhancement areas in the corticomedullary region. These infarcted areas are typically wedge-shaped with extension to the renal capsule. In patients with total occlusion of the main renal artery, the whole kidney may appear as a completely non-enhancing organ with only capsular enhancements due to collateral capsular circulation. This capsular enhancement is also known as the “cortical rim” sign [38].

### Spontaneous dissection of the renal artery

Acute renal artery dissections are mostly traumatic in origin. However, despite being rare, spontaneous renal artery dissection (SRAD) has also been reported in the literature [39]. Among the predisposing factors to SRAD, atherosclerosis, intimal fibroplasia, severe hypertension, Marfan syndrome, and Ehlers–Danlos syndrome have been mentioned [40].

**Fig. 12:**
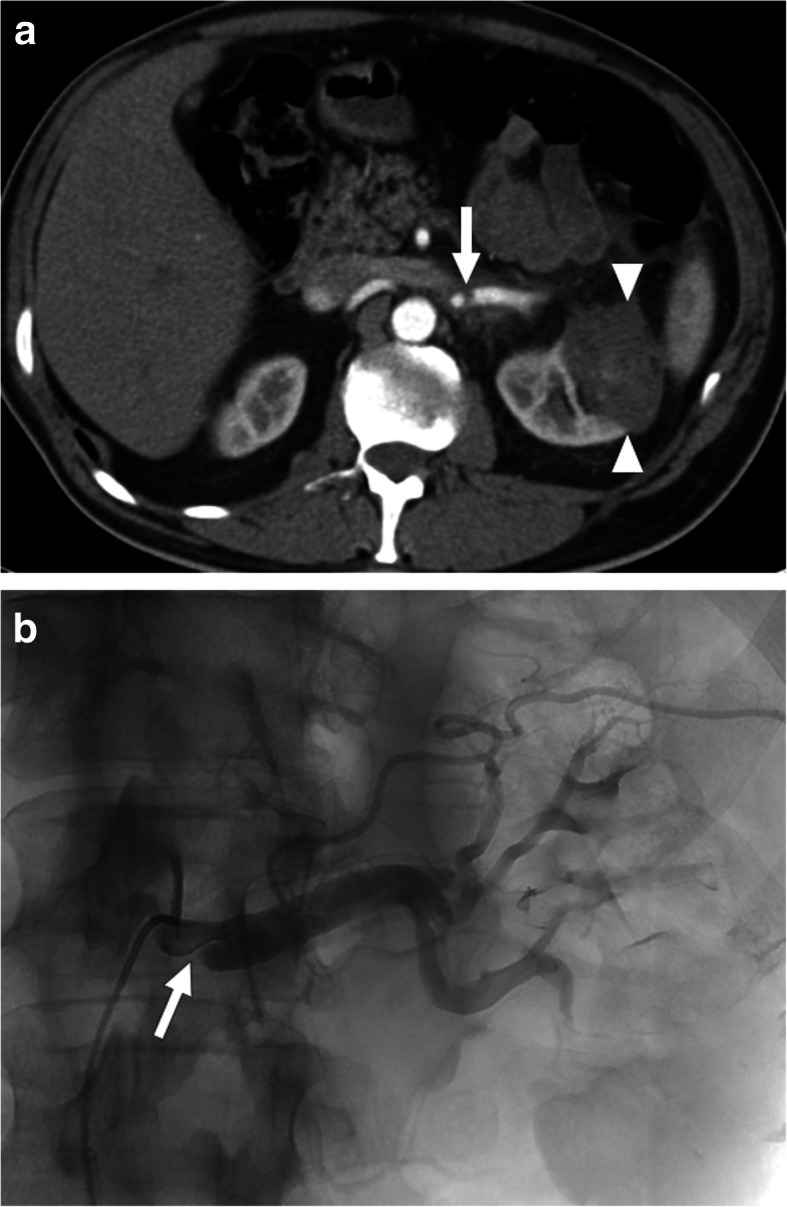
Spontaneous renal artery dissection. 44-year-old male with known Marfan syndrome presented to ED with acute onset severe left flank pain and massive hematuria. US examination showed enlarged left kidney with decreased color Doppler signal in the upper pole of the kidney parenchyma. **a** Arterial phase CT angiography image showed flap in the proximal third of the left renal artery (arrow). Also, note is made of large infarct in the upper pole parenchyma (arrowheads). **b** Emergent catheter angiography confirmed the presence of the intimal flap (arrow)

Most patients with SRAD present with non-specific symptoms such as flank pain, hematuria, hypertension, and headache. SRAD has been reported mostly in the right renal artery in young and middle-aged men with newly diagnosed hypertension who also have underlying atherosclerosis or FMD. Early recognition and intervention are critical for a favorable outcome. Several other clinical situations including thromboembolism, renal vein thrombosis, and renal infections may also present with similar symptoms [40]. CTA is an excellent tool in these patients for differential diagnosis. In arterial phase imaging, direct visualization of the vascular flap may be possible. Associated findings such as renal artery thrombosis and parenchymal infarcts may also be easily detected with CTA (Fig. 12).

Clinical management of renal artery dissections is controversial and immediate nephrectomy, nonoperative management, and surgical/endovascular revascularization have all been proposed as potential treatment approaches [41]. Salvage rates have been reported to be low after surgical revascularization with a very high rate of recurrent arterial thrombosis [42].

### Renal artery aneurysms and pseudoaneurysms

**Fig. 13:**
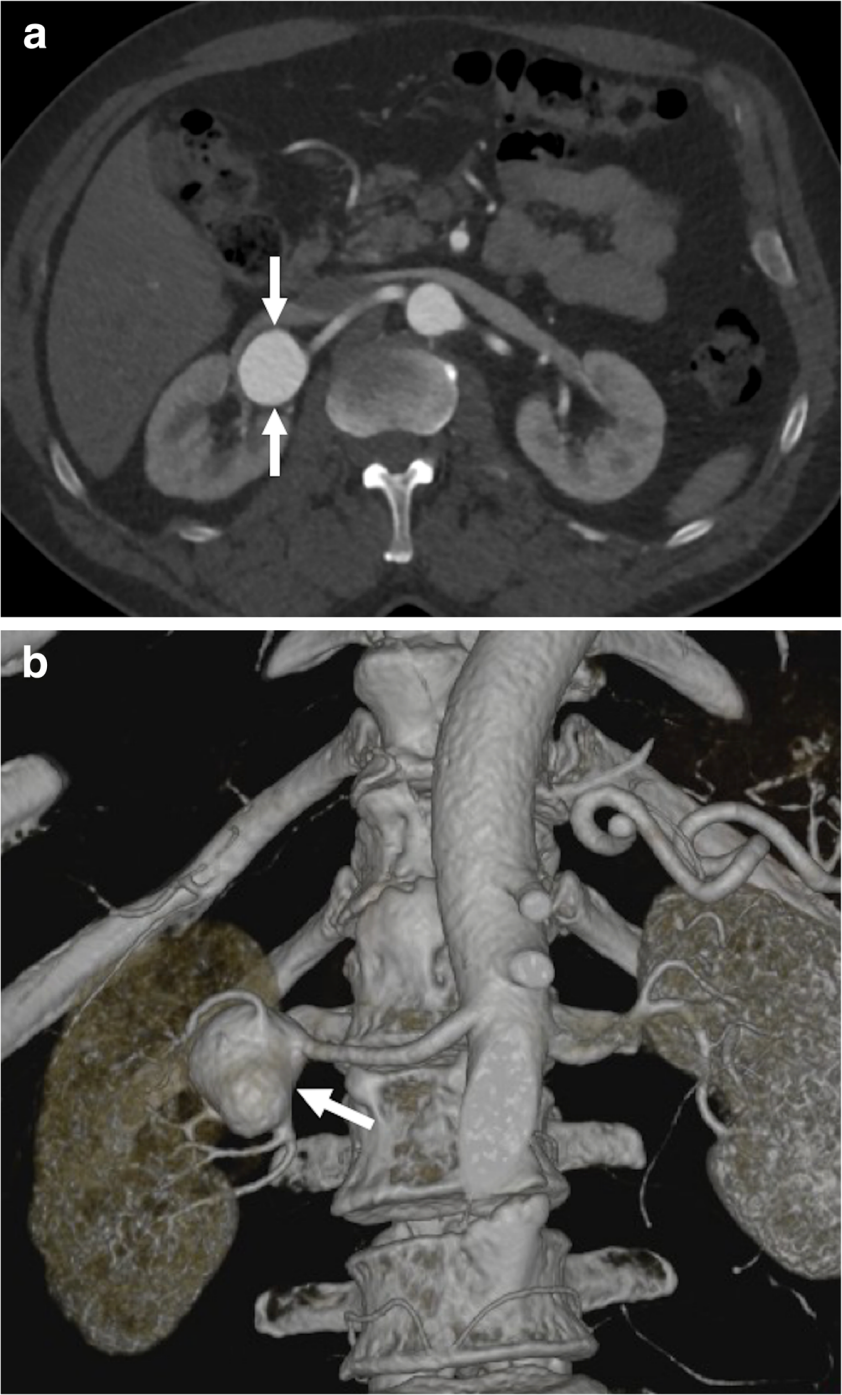
Renal artery aneurysm. A 56-year-old male with no past medical history presented to ED with right flank pain. Preliminary US study showed a large cystic mass within the right renal hilum. **a** Axial arterial phase CT image shows a large renal artery aneurysm (arrows) with no evidence of rupture. **b** Reformatted volume-rendered image from the same study clearly demonstrates the aneurysm arising from the distal portion of the right renal artery (arrow). The patient was subsequently treated with elective surgery

Renal artery aneurysms (RAAs) are very rare with a reported incidence of 0.03% to 0.09% on autopsy studies. However, with the common use of imaging, the incidence rate increases, with an estimated incidence rate of 1% [43]. The majority of the RAAs are detected in asymptomatic patients. Atherosclerosis and fibromuscular dysplasia are the most common underlying reasons for RAA formation [44]. Hereditary intrinsic collagen deficiencies may also be worked up in select patients as potential underlying risk factors. Hypertension was reported to be the most common presenting symptom (90%) which is thought to occur secondary to altered blood flow due to kinking or twisting of the renal artery with subsequent increased renin secretion induced hypertension [45, 46]. Rupture of the aneurysm is the most dramatic presentation which may cause life-threatening internal bleeding with a mortality rate of 10% [47]. Unruptured large renal artery aneurysms may also cause severe flank pain and may mimic other more common kidney problems such as pyelonephritis or nephrolithiasis. Pregnancy, polyarteritis nodosa, and a history of liver disease are the most commonly encountered risk factors for spontaneous rupture [48]. On cross-sectional imaging, RAAs appear as a contrast filling (may also appear as partially thrombosed/calcified), nodular structure along the course of the renal artery (Fig. 13). Reformatted images may better delineate the anatomic orientation of the aneurysms which may be critical for endovascular or surgical planning. Treatment is indicated in incidentally detected RAAs which are larger than 2 cm in diameter [45].

**Fig. 14:**
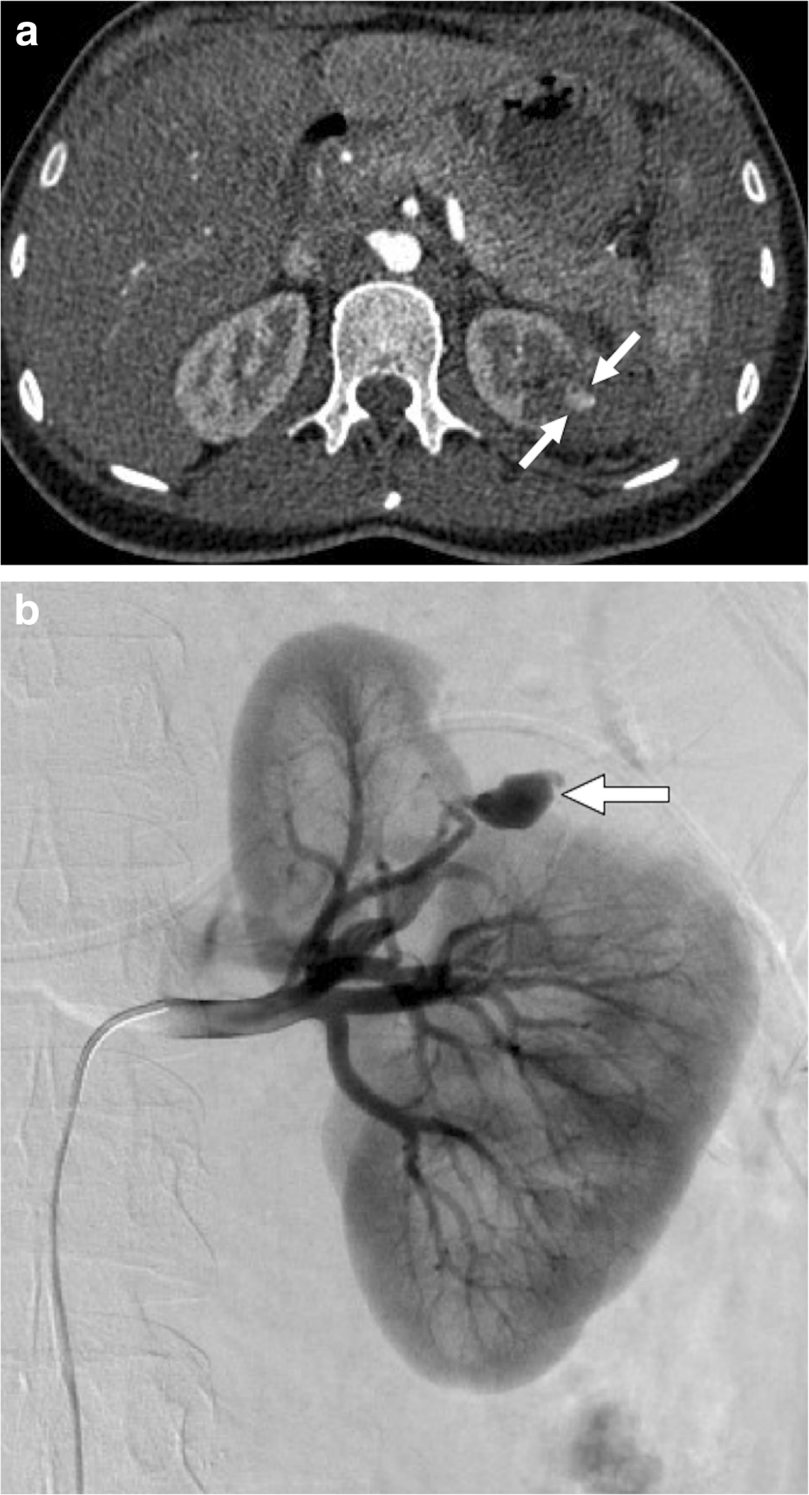
Renal artery pseudoaneurysms. A 36-year-old female underwent partial nephrectomy for low-grade RCC presented to ED with left flank pain and hematuria 12 days after the surgery. **a** Axial post-contrast arterial phase CT image shows a small pseudoaneurysm (arrows) at the resection bed. There was also a hematoma surrounding the pseudoaneurysm. **b** Selective left renal artery catheter angiography clearly demonstrated the pseudoaneurysm (arrow). The pseudoaneurysm was successfully treated with detachable coils in the same session

Renal artery pseudoaneurysms (RAPs) arise from arterial injuries and with subsequent loss of vessel wall integrity. Surgical and percutaneous procedures in addition to penetrating trauma and infectious causes are among the common underlying causes of RAP formation [45]. The sac is contained by the media or adventitia of the vessel or by the perivascular tissues. Through the neck of this perfused sac, the pseudoaneurysm is in direct communication with the arterial lumen. Vasculitis may also cause several pseudoaneurysms within the renal parenchyma. In contrast to the rare occurrence of spontaneous rupture in RAAs, RAPs may spontaneously rupture more frequently [45]. CT angiography is the preferred modality for diagnosis. Contrast filling saccular structure is a common finding in RAP. Endovascular treatment is the preferred approach for treatment of renal pseudoaneurysm but surgical intervention may also be indicated in certain patients (Fig. 14).

### Renal arteriovenous fistulas

Renal arteriovenous fistulas (AVFs) are typically caused by penetrating or blunt traumas and iatrogenic procedures such as surgery and open/percutaneous biopsy. After kidney biopsies, the reported rates of renal AVFs are 7.4–11% [49]. Renal AVFs forming after biopsy typically resolve spontaneously but massive life-threatening hematuria may also be detected in certain patients [49]. Endovascular intervention is the preferred treatment approach for these patients.

**Fig. 15:**
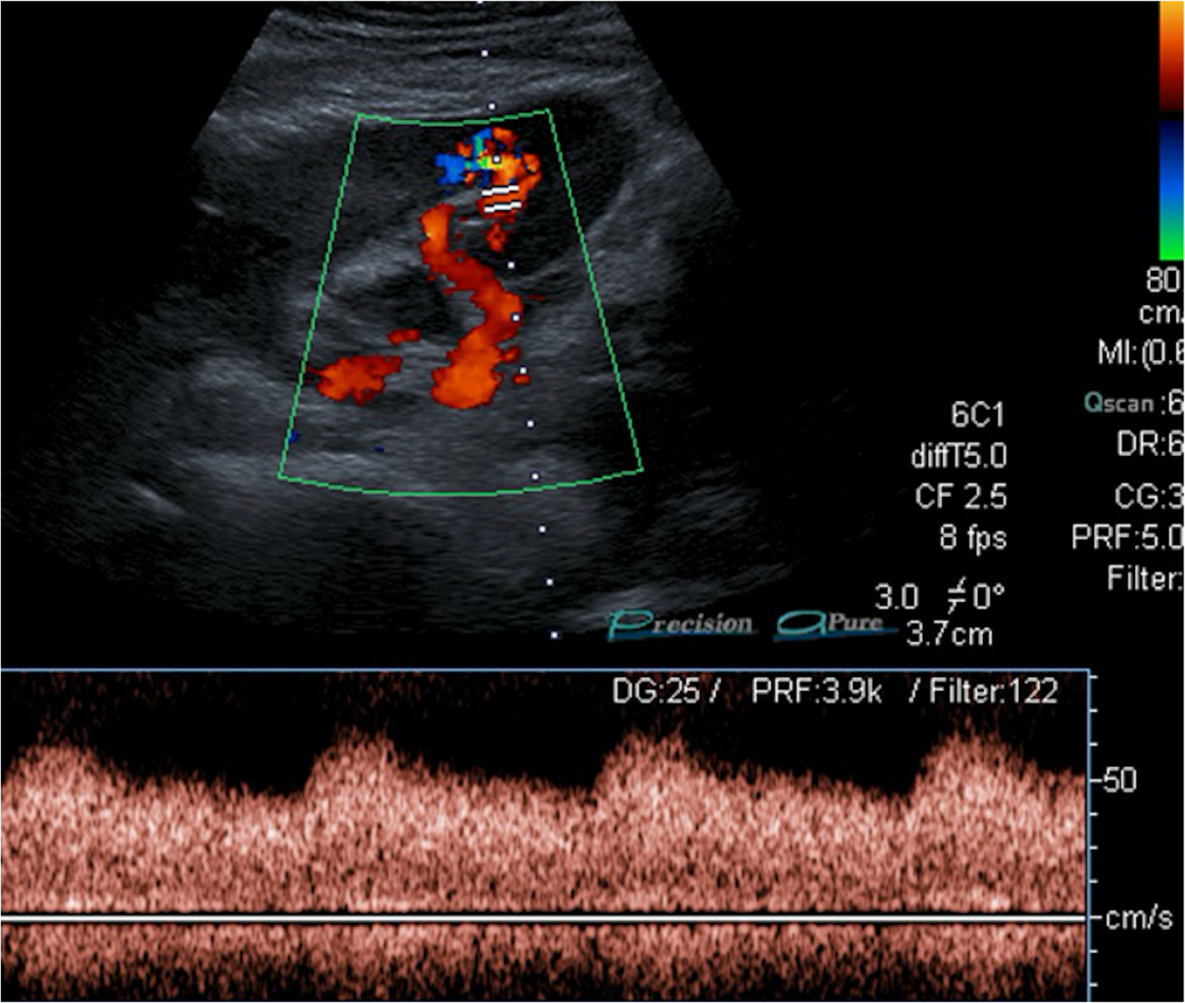
Renal arteriovenous fistula. A 28-year-old female with a history of renal transplantation recently underwent percutaneous renal biopsy for suspected graft rejection. The patient experienced hematuria the day after the biopsy. Doppler US evaluation of the graft kidney demonstrates aliasing in the lower pole parenchyma suggestive for high velocity disturbed flow. The flow waveform was also suggestive for arterialized venous flow with reduced systolic-diastolic difference. The patient underwent CO_2_ angiogram with subsequent successful coil embolization

**Fig. 16:**
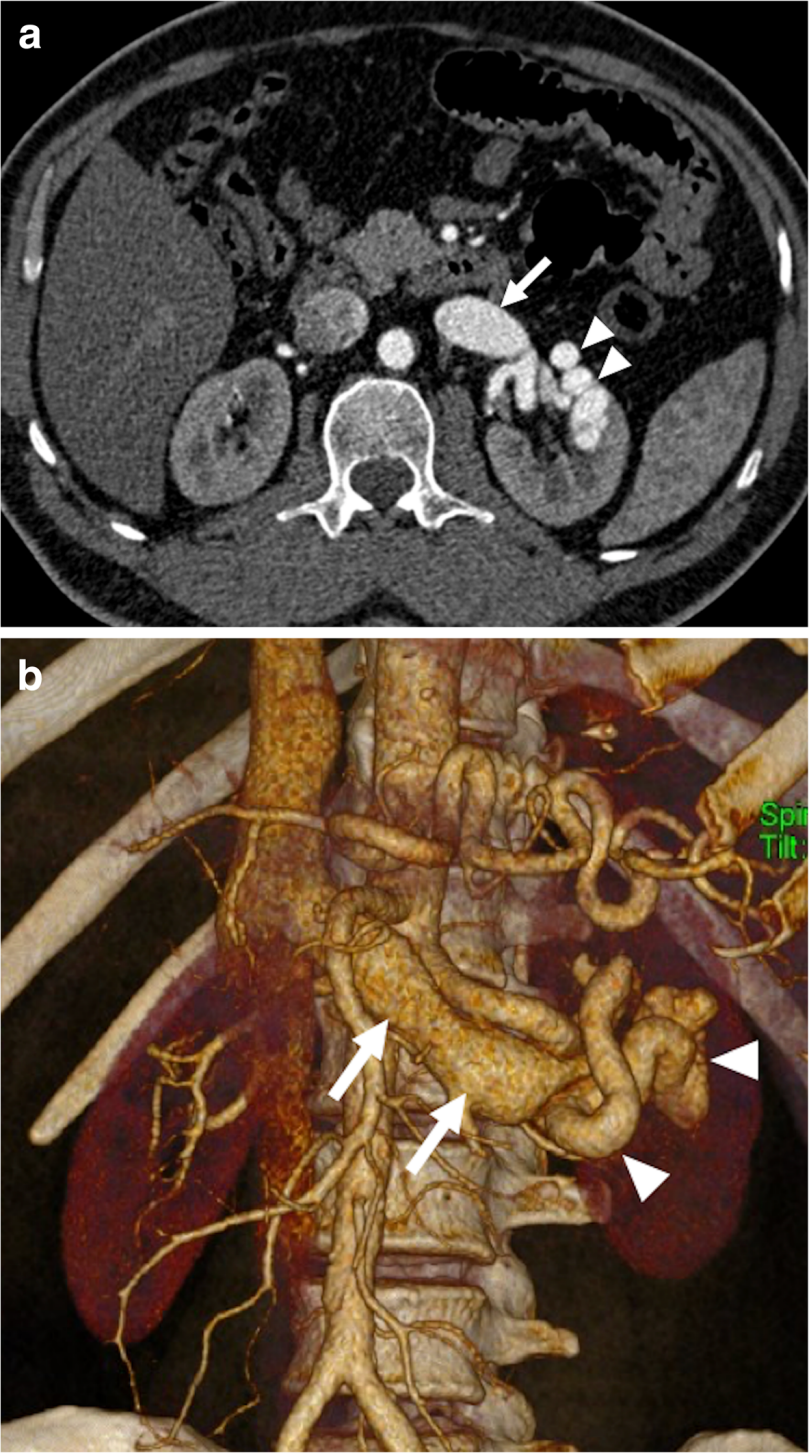
Renal arteriovenous fistula. A 53-year-old male patient with a known history of nephrotic syndrome of unclear etiology presented to ED with recent-onset left flank pain and hematuria 2 months after percutaneous kidney biopsy. **a** Arterial phase axial plane post-contrast CT image showed early contrast filling of the dilated left renal vein (arrow). Also, note is made of associating ectatic venous structures within the renal hilum (arrowheads). **b** Coronal volume rendered image shows dilated left renal vein (arrows) with tortuous fistula tract having fusiform dilations (arrowheads) within the renal hilum. The patient was subsequently treated with surgical ligation and resection

Doppler US is commonly the first imaging modality used for diagnosis. Small renal AVFs may not be visualized on gray-scale US; however, large AVFs may be detected as a cystic or tubular anechoic mass. Color Doppler US may confirm the vascular nature of this mass and spectral analysis demonstrates increased flow velocity and arterialized flow within the renal vein (Fig. 15). Early contrast filling of the renal veins with ectatic vessels at the AVF site are characteristic imaging findings on CT and MR studies [49] (Fig. 16).

### Renal vein thrombosis

**Fig. 17:**
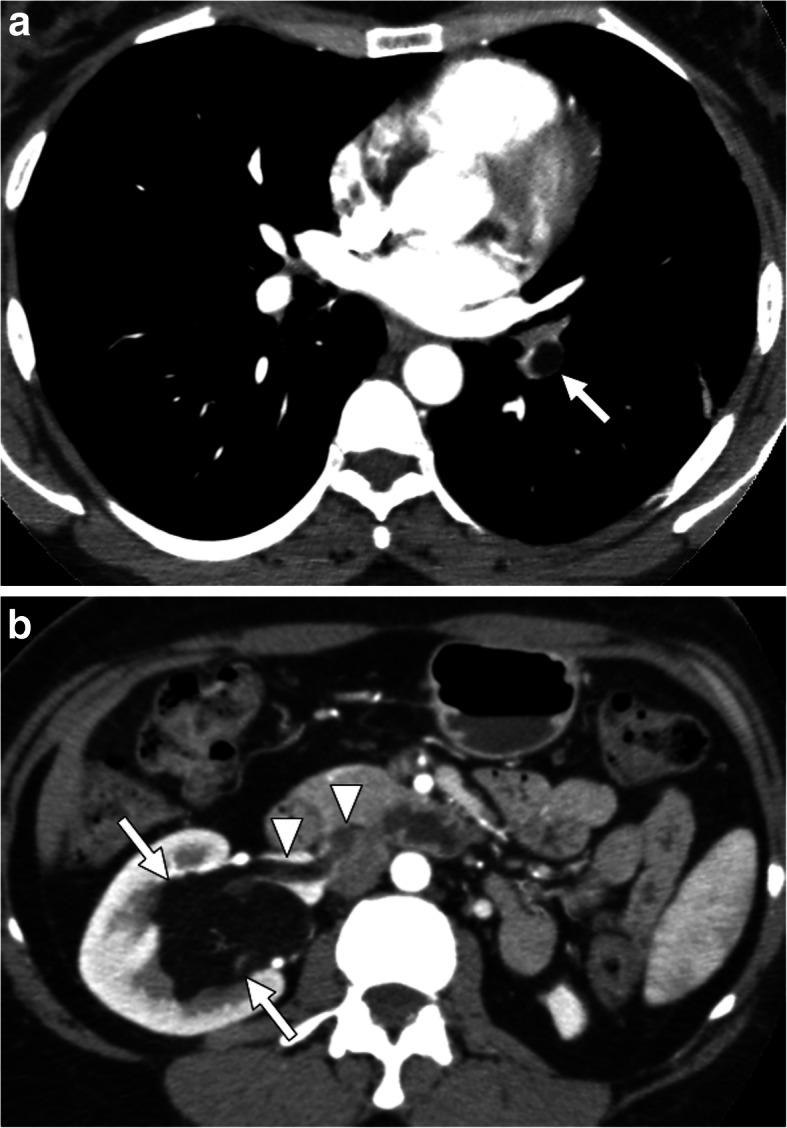
Tumor thrombus from renal AML. A 42-year-old man with no past medical history now present with acute shortness of breath and hematuria. **a** Pulmonary CT angiography demonstrates an endoluminal thrombus of macroscopic fat density (arrow) within the left lower lobe pulmonary artery. **b** Subsequent abdominal CT detected angiomyolipoma (arrows) in the right kidney with tumoral thrombus (arrowheads) in the right renal vein and IVC

Renal vein thrombosis (RVT) is, by definition, thrombotic occlusion of unilateral or bilateral renal veins. Acute RVT is rare in adults and is most commonly detected in neonatal age group. The clinical progress is typically insidious and, in adults, it typically occurs in patients with nephrotic syndrome and renal cell cancer. The pathophysiology is similar to other forms of venous thrombosis with 3 distinct predisposing factors: stasis, endothelial damage, and hypercoagulability [50]. In adults, it is mostly unilateral. Clinical symptoms can be variable ranging from asymptomatic to massive hematuria and the symptoms are mostly related to rate and extent of thrombus formation [51] (Fig. 17).

**Fig. 18:**
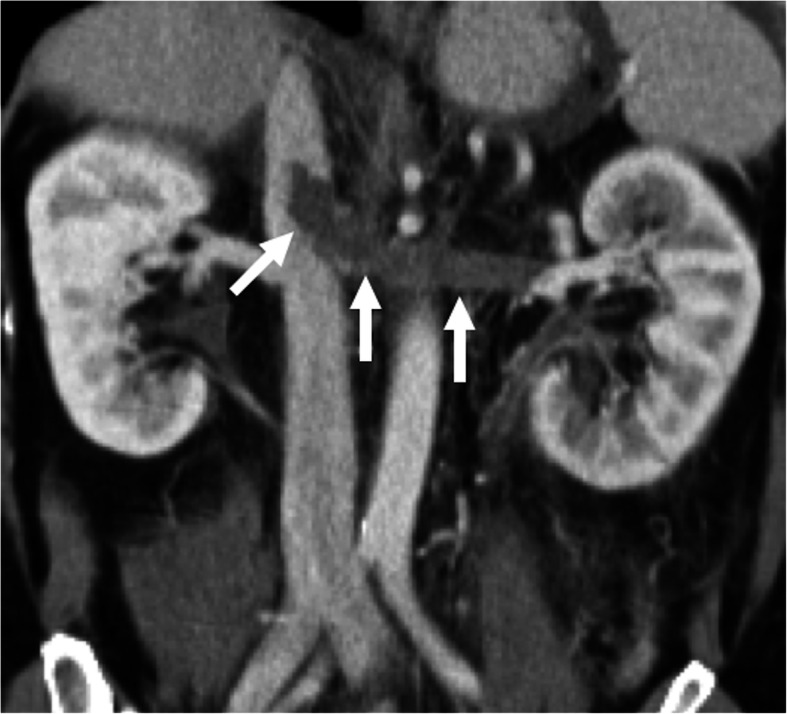
Renal vein thrombosis. A 38-year-old woman with known antiphospholipid antibody syndrome (APS) presents to ED with severe left flank pain and gross hematuria. Coronally reformatted post-contrast CT image demonstrates a large thrombus (arrows) in the left renal vein with minimal extension into the IVC

Early imaging and diagnosis may play an important role for averting the effects of parenchymal ischemia. On US, the detection of thrombus on gray-scale or color Doppler US is diagnostic. Parenchymal changes may be better observed with CT or MRI. The luminal thrombus may be better appreciated on reformatted images in different planes (Fig. 18). The kidneys and other neighboring organs may also be carefully evaluated to detect a potential underlying neoplasia.

### Nutcracker syndrome

Nutcracker syndrome (NCS), also known as the “left renal vein (LRV) entrapment syndrome,” is characterized by severe luminal stenosis of the left renal vein between the superior mesenteric artery (SMA) and the abdominal aorta. This compression increases the endoluminal pressure in the LRV and subsequent development of the collateral veins [52]. There is normally a wide angle between the retroperitoneal adipose tissue and the third part of the duodenum, with the normal angle of 90° between the SMA and the abdominal aorta [53]. In patients with diminished retroperitoneal fat and thin habitus, this aorto-mesenteric angle (AMA) may decrease, causing subsequent LRV compression between these vessels.

Clinical presentation is highly variable ranging from asymptomatic microscopic hematuria to macroscopic hematuria. Gross hematuria may be rarely seen in patients with nutcracker syndrome and should be considered among the rare causes of this clinical occurrence. Left flank pain, varicocele, pelvic congestion, or ovarian vein syndrome may be counted among other symptoms or complications [53–55]. From a pathophysiological standpoint, hematuria is attributed to the rupture of dilated submucosal vessels, which develop due to venous hypertension, into the calyceal fornices [56].

**Fig. 19:**
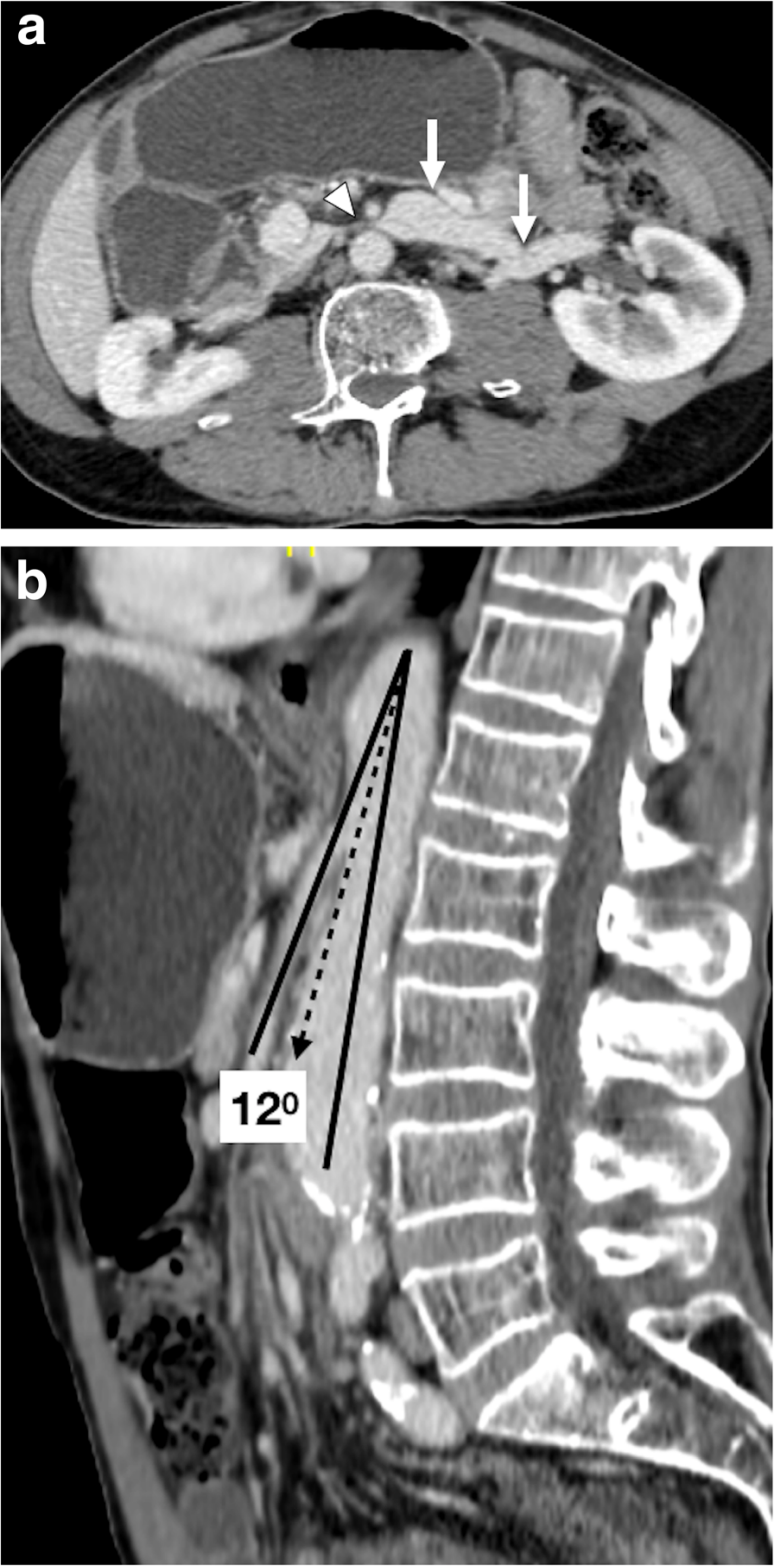
Nutcracker syndrome. A 67-year-old male with known aortic aneurysm presented to ED with gross hematuria. The sonographic examination of the abdomen was unremarkable. **a** Axial plane venous phase CT image shows significant distension of the left renal vein (arrows) with severe and abrupt narrowing in the preaortic area (arrowhead). **b** The aortomesenteric angle was measured to be 12°. There was no evidence of urinary stone disease or TCC in the same exam. The patient was placed on conservative therapy

CT is the main study for evaluation for NCS. The acute narrowing of the LRV between the SMA and the abdominal aorta, the beak sign, with proximal dilation of the LRV is the main finding, with a sensitivity of 91.7% and specificity of 88.9%. The ratio of LRV diameters at the kidney hilum and aortomesenteric regions is typically more than 4.9 (Fig. 19) [56]. AMA is the other helpful finding for diagnosing NCS. An angle of less than 41° is 100% sensitive and 55.6% specific for NCS [57]. The beak angle may be measured by drawing two lines along the anterior and posterior walls of LRV as it courses immediately underneath the SMA to the point of narrowing of the LRV. A beak angle of more than 32° is 83.3% sensitive and 88.9% specific for NCS [56].

### Ovarian vein thrombosis

Ovarian vein thrombosis (OVT) is a rare clinical condition which typically associates with pregnancy, malignancy, inflammatory bowel disease, and pelvic inflammatory disease [58]. The reported rate is around 0.05% to 0.16% and is most commonly seen in early postpartum period. Right gonadal vein is more commonly affected (around 90%) than its left counterpart and incompetent valves, longer length, and dextrorotation of the gravid uterus may be counted among the contributors to this condition. Ovarian vein diameters increase up to threefold during pregnancy which may lead to stasis and subsequent venous thrombosis [58].

The most commonly encountered clinical presentation is the triad of pelvic pain, fever, and right-sided abdominal mass. Tachypnea, flank pain, ileus, nausea, and vomiting may also be seen in affected patients. Blood cultures are rarely positive and symptoms most commonly occur in the first 4 weeks of the postpartum period [58].

**Fig. 20:**
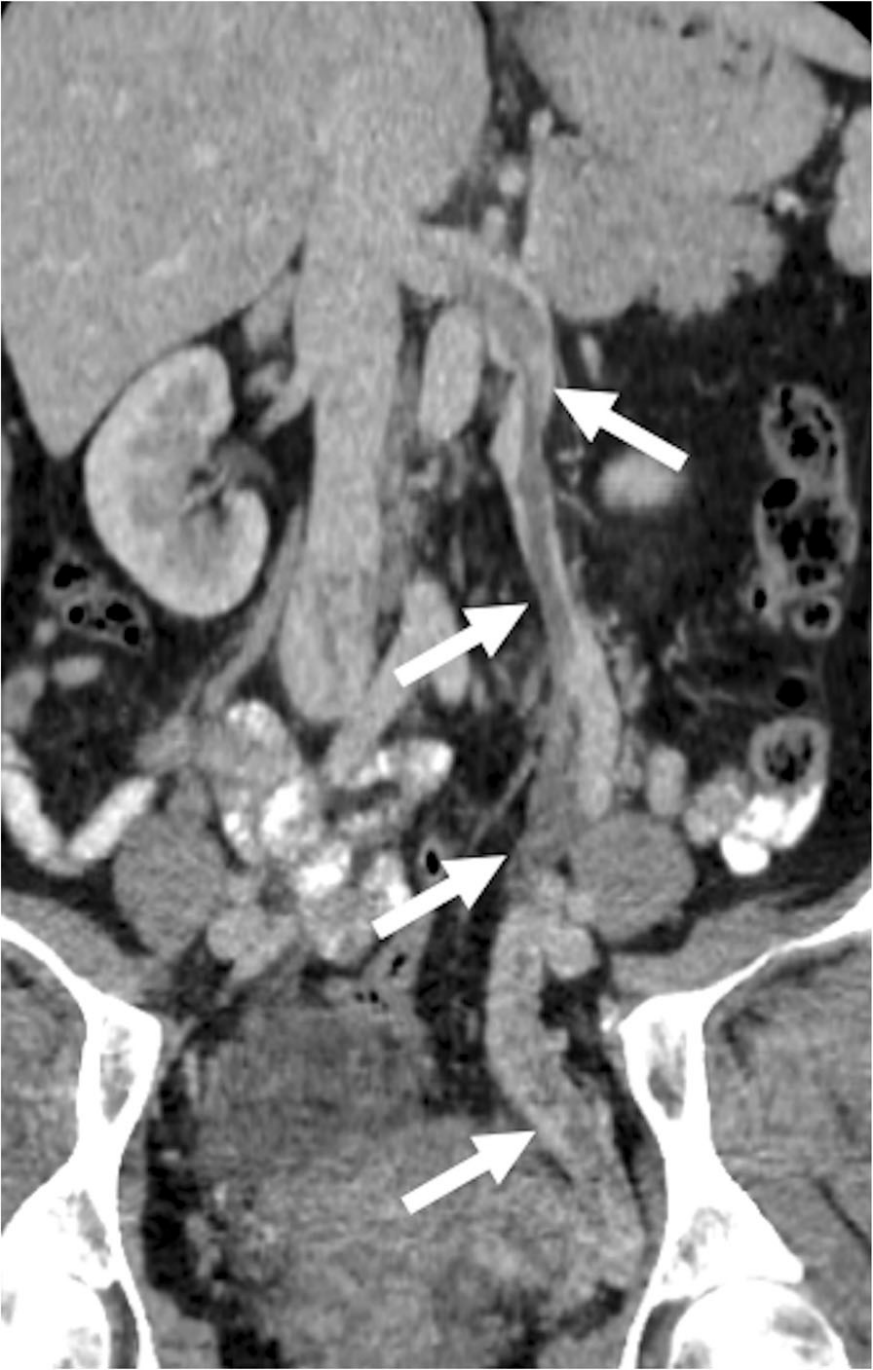
Ovarian vein thrombosis. A 44-year-old female presented to ED with fever and abdominal pain 13 days after laparoscopic myomectomy. Coronally reformatted post-contrast CT image demonstrates acute thrombus (arrows) within the left gonadal vein with extension into the left renal vein

Imaging diagnosis may be difficult with US as ovarian veins lie deep in the retroperitoneum shielded by intraperitoneal organs. CT is the most commonly used modality in suspected patients and diagnosis may be made promptly with CT. Reformatted images are extremely helpful. The thrombosed ovarian vein appears as an enlarged vessel with an endoluminal filling defect (Fig. 20). Anticoagulation and antibiotics are the mainstays of treatment.

## Superior mesenteric artery

Acute mesenteric ischemia (AMI) may be a devastating disorder with a grave prognosis. Superior mesenteric artery (SMA) embolism and thrombosis are the most common causes of AMI. However, severe hypotension or metabolic shock may also cause nonocclusive mesenteric ischemia (NOMI) in patients with patent SMA due to severe reduction and redistribution of the mesenteric arterial flow [59].

### Acute occlusive thrombotic disease of the superior mesenteric artery

SMA is the feeding artery of the midgut and is the second major branch of the abdominal aorta. This vessel is particularly vulnerable to embolism from distant sources due to the acute take-off of the vessel origin from the abdominal aorta. Occlusive thrombotic disease of the SMA may have grave clinical consequences. The clinical symptoms are mostly related to abdominal pain [60]. Arterial embolus is the main cause (40–50%) followed by endoluminal thrombosis (15–30%). Non-occlusive mesenteric ischemia is also seen in 10–20% of the cases with no obstructive thrombi in the SMA [59].

SMA embolus generally originates from the left atrium as a result of atrial fibrillation in elderly patients. Thrombi and large emboli may occlude the proximal part of SMA and ostia of its major branches; however, distal branches may also be affected with smaller emboli. The majority of these emboli typically lodge within the proximal 3–10 cm of the SMA; therefore, proximal jejunum and colon may be spared [61]. The presence of concurrent emboli and infarction in other vascular territories (e.g., spleen and kidney) may suggest a proximal embolic source rather than thrombosis. The presence of extensive atherosclerotic plaques may be regarded as a warning sign for acute thrombosis.

Multidetector CT of the abdomen with IV contrast injection is the first-line modality. Both arterial and venous phase images should be acquired to evaluate both arterial and venous mesenteric vessels. Isotropic reformatted images and maximum intensity projections images in several different planes may be extremely helpful for evaluating the extension of the vessel involvement [59].

**Fig. 21:**
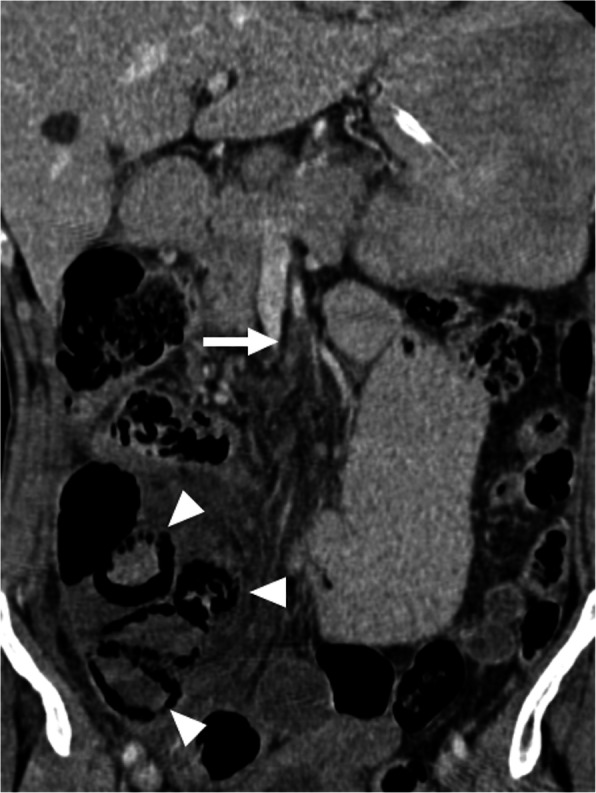
SMA thrombosis. A 65-year-old male with a previous history of surgically treated intestinal gastrointestinal stromal tumor presented with abdominal pain, distension and tenderness. Coronally reformatted post-contrast CT image demonstrates thrombotic occlusion of SMA (arrow) 4–5 cm distal to its origin. Also, note the presence of air within the walls of the small bowel segments consistent with pneumatosis intestinalis (arrowheads). Surgery confirmed infarcted ileal segments and the patient underwent intestinal resection

Emboli or thrombi typically appear as low-attenuating filling defects in the mesenteric vessels. The associated features of intestinal ischemia should also be searched for (Fig. 21). Persistent arterial occlusion and subsequent transmural bowel wall infarction may destroy intramural nerves and intestinal musculature. This causes infarcted bowel segments to dilate with extreme wall thinning known as “paper-thin wall” and even perforation. A high-attenuating bowel wall at precontrast images may indicate a hemorrhagic infarction, whereas mild bowel wall thickening and hyperattenuating bowel wall at postcontrast images may be observed in reversible ischemia, congestion or reperfusion [59, 61].

NOMI is usually a consequence of mesenteric vasospasm and contraction [61]. It typically is observed in critically ill patients in intensive care units. Sepsis, hypovolemia, and the extensive use of vasoconstrictive agents may all give rise to this clinical condition. The SMA is typically patent in these patients but may appear as severely constricted vessels with severely narrowed lumens. Ischemic changes are usually broad and include both small intestine and colon but typically are more prominent in the terminal ileum and the right colon. Affected intestinal loops may not show enhancement on CT with intervening normally enhancing bowel loops [59, 62].

### Superior mesenteric artery dissection

**Fig. 22:**
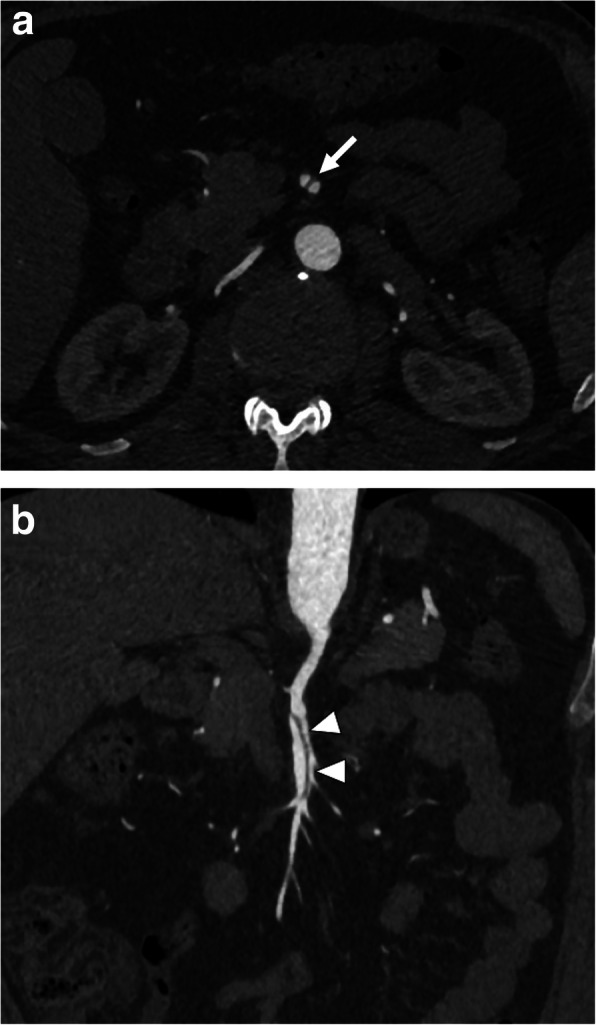
SMA dissection. A 59-year-old male with known COPD presented with severe diffuse abdominal pain. **a** Axial plane arterial phase CT image shows a linear hypodense thin structure suggestive of an intimal flap within the SMA lumen (arrow). **b** Coronally reformatted post-contrast arterial phase CT image demonstrates long segment dissection (arrowheads) within the SMA with no extension into the abdominal aorta. The findings were found to be suggestive for isolated SMA dissection

SMA dissections can be an isolated event or combined with aortic dissection. Combined SMA dissection is more common and is secondary to an aortic flap extending into the SMA lumen. Spontaneous SMA dissection is a rare clinical phenomenon that occurs without associating aortic flap [63]. Even though it is a rare disease, its detection is becoming more common with the ever-increasing use of cross-sectional imaging (Fig. 22). Isolated SMA dissection is more common in patients between the ages of 50–70 [64]. The flap within the vessel wall may narrow or completely occlude the vessel lumen and may cause mesenteric ischemia. SMA dissections may be clinically silent; however, emergent presentation with acute abdominal pain, nausea, vomiting, and bloody stools may also be seen [65]. The false lumen may appear as thrombosed with low attenuation and the false lumen mostly lies along the greater curvature of the SMA, and the true lumen is along the lesser curvature [66]. Treatment can be observational with supportive treatment and surgical/endovascular interventions.

### SMA aneurysm

SMA aneurysm is the third most common type of non-traumatic visceral artery aneurysms and accounts for 5.5% of all visceral artery aneurysms. They are most commonly detected within the first 5 cm of SMA and may be saccular or fusiform in shape. Infection, dissection, atherosclerosis, and pancreatitis are among the common causes [31, 67]. Unlike other visceral aneurysms, SMA aneurysms are often symptomatic with common symptoms of abdominal pain, nausea, and vomiting or hemorrhage. The patients are at high risk for bowel ischemia and aneurysm rupture may occur in almost 50% of the patients, especially in non-calcified aneurysms or aneurysms larger than 2 cm in diameter [31].

**Fig. 23:**
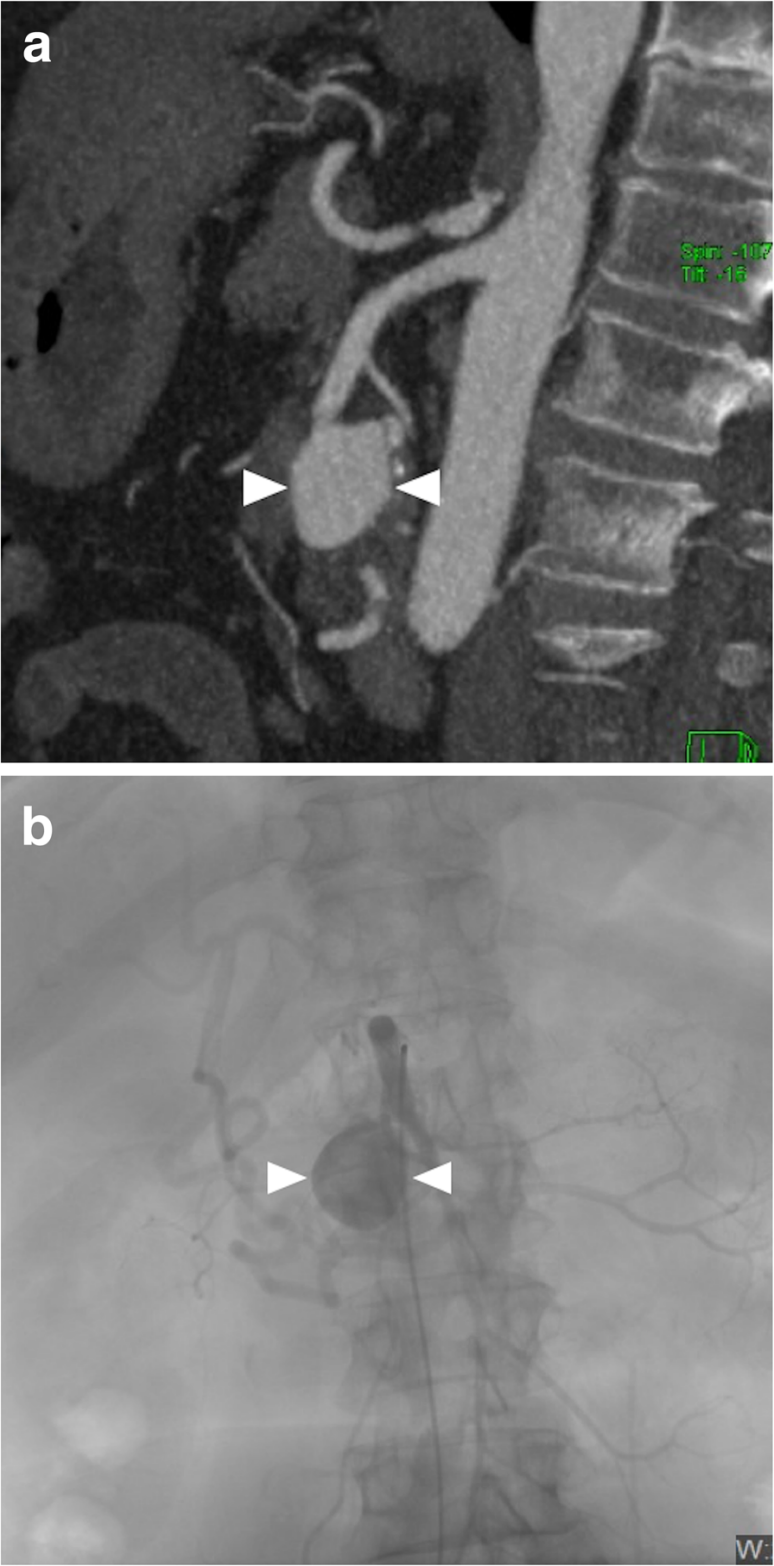
SMA aneurysm. A 62-year-old female with a past medical history of hypertension and type 2 diabetes now presents to ED with diffuse abdominal pain. **a** Sagittal plane reformatted post-contrast CT image demonstrates a large saccular aneurysm (arrowheads) originating from the SMA trunk. **b** Catheter angiography confirmed the presence of the aneurysmal sac (arrowheads) originating from the SMA. The aneurysm was successfully embolized with coils in the same session

CT angiography is the preferred imaging modality for detecting SMA aneurysms. The aneurysm morphology is variable and may appear as either saccular or fusiform in shape (Fig. 23). Associated atherosclerotic wall calcifications may be seen in certain patients. A mural thrombus, if present, may cause bowel ischemia by distal embolization [31].

Due to the high incidence of rupture and complications, treatment is indicated for most SMA aneurysms regardless of their size [31]. Treatment options depend on the patient’s condition (hemodynamic status, surgical risk), location, and size of the aneurysm. Endovascular and surgical approaches may be used in treatment [67].

### Superior mesenteric artery syndrome

SMA syndrome is caused by compression of the third part of the duodenum between SMA and the abdominal aorta. The duodenum is surrounded by adipose tissue, which serves as a cushion against external compression [68] . In patients with severe weight loss and low body mass index, the fat plane in the aortomesenteric plan may be effaced and duodenum may entrap between SMA and the abdominal aorta. SMA syndrome is uncommon and its prevalence was reported to be 0.013–0.3% on fluoroscopic studies [69] with a slight female preponderance of (64–66%) [68]. Post-prandial and intermittent abdominal pain with bouts of vomiting and nausea due to intestinal obstruction are the most common presenting symptoms. As the findings are non-specific, high index of clinical suspicion is mandatory for diagnosis.

**Fig. 24:**
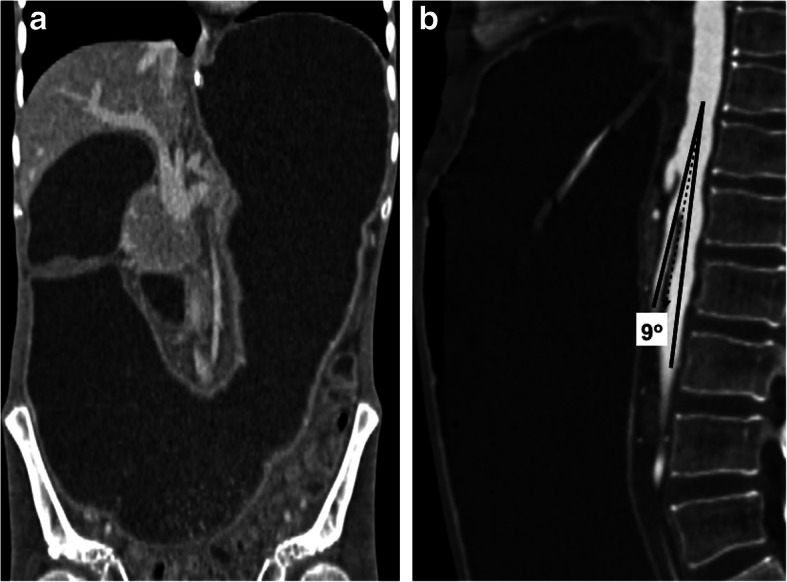
SMA syndrome. A 37-year-old female with no past medical history presented to ED with severe nausea and vomiting. Physical examination revealed a significantly distended abdomen. **a** Coronally reformatted post-contrast CT image demonstrates a severely distended stomach and proximal duodenum. **b** Sagittal post-contrast CT image showed markedly decreased aortomesenteric angle and aortomesenteric distance. Findings were found to be consistent with SMA syndrome. Endoscopic study confirmed this diagnosis and revealed no evidence of obstructing endoluminal mass

CT is the most commonly used modality for diagnosis. Aortomesenteric angle measurement is crucial for diagnosis and an angle less than 22° and the aortomesenteric distance of less than 8 mm (the distance between the anterior wall of the abdominal aorta and the posterior wall of the SMA) (Fig. 24). The normal aortomesenteric distance is between 10 and 28 mm and is measured at the level of the duodenum as it traverses between the abdominal aorta and the SMA [56]. The detection of dilated stomach and the duodenum with abrupt cessation of duodenal distension as it traverses underneath the SMA [70].

## Celiac artery

### Isolated celiac artery dissection

Isolated spontaneous celiac artery dissection, without associating aortic dissection, is a very rare clinical event [71]. Due to its rarity, the clinical course is unclear; however, most of the reported patients have a benign course [72]. It is primarily treated conservatively with blood pressure control and antithrombotic measures. It is typically seen middle-aged male patients and sudden onset abdominal pain is the main presenting symptom [73].

**Fig. 25:**
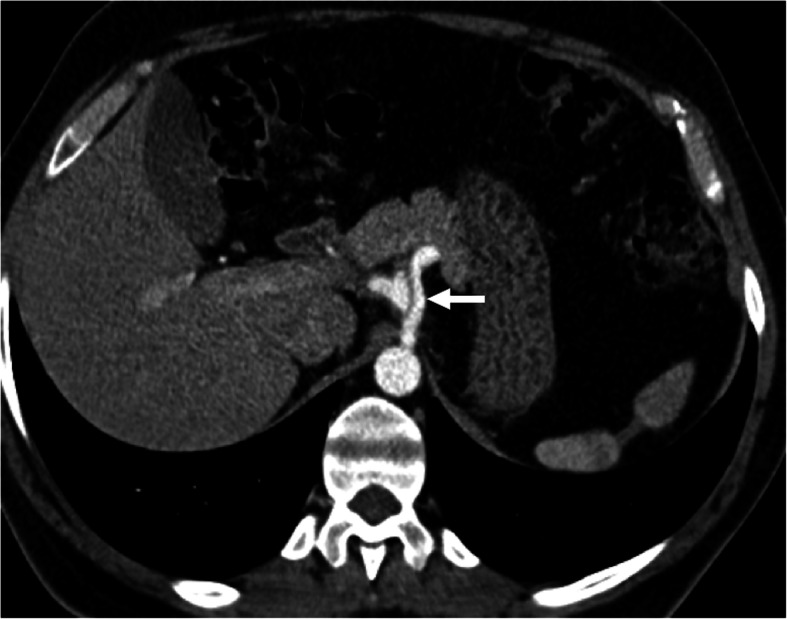
Celiac artery dissection. A 37-year-old male with known polyarteritis nodosa presented to ED with excruciating epigastric pain. Axial plane post-contrast CT image demonstrates the intimal flap in the celiac trunk consistent with isolated dissection (arrow). There was no associating abdominal aortic dissection

**Fig. 26:**
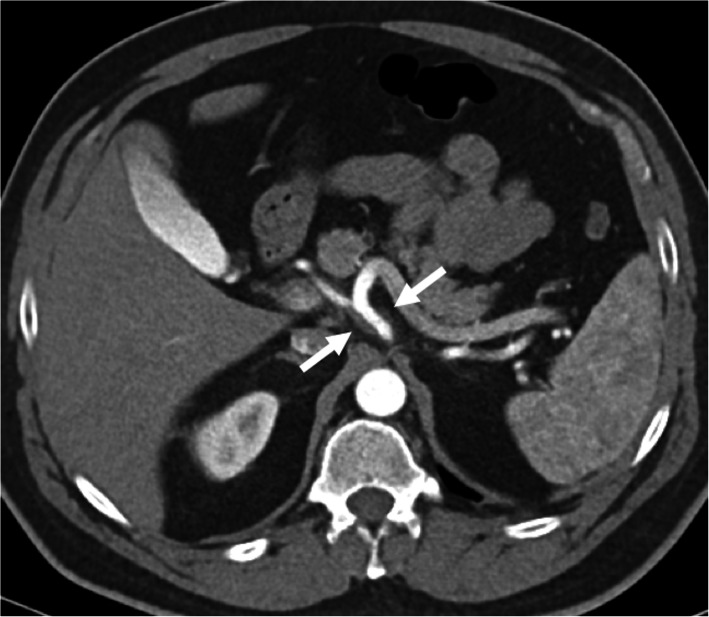
Celiac artery dissection. A 47-year-old heavy smoker male with known hypertension presented to ED with sharp epigastric pain. Axial plane post-contrast image shows thickening of the celiac artery wall (arrows) suggestive for intramural hematoma and celiac artery dissection. The patient responded well to conservative medical treatment and follow-up imaging study confirmed the regression of intramural hematoma (not shown)

CT and MRI may both be used for diagnosis but due to its versatility; contrast-enhanced CT appears to be more pertinent for diagnosis. On CT, dissection flap may be directly seen; however, intramural hematoma with/without associating penetrating ulcer may also be detected [73]. Dissection flap is seen as a linear, thin hypodense structure (Fig. 25). Intramural hematoma without associating flap is characteristically seen as a wall thickening with associating perivascular soft tissue structure (Fig. 26). Penetrating ulcers are seen as contrast filling outpouchings from the vessel wall.

### Celiac artery thrombosis

Celiac artery thrombosis is an unusual cause of acute abdomen. Early diagnosis is crucial as the consequences may cause high morbidity and mortality when the diagnosis and treatment are delayed [74].

**Fig. 27:**
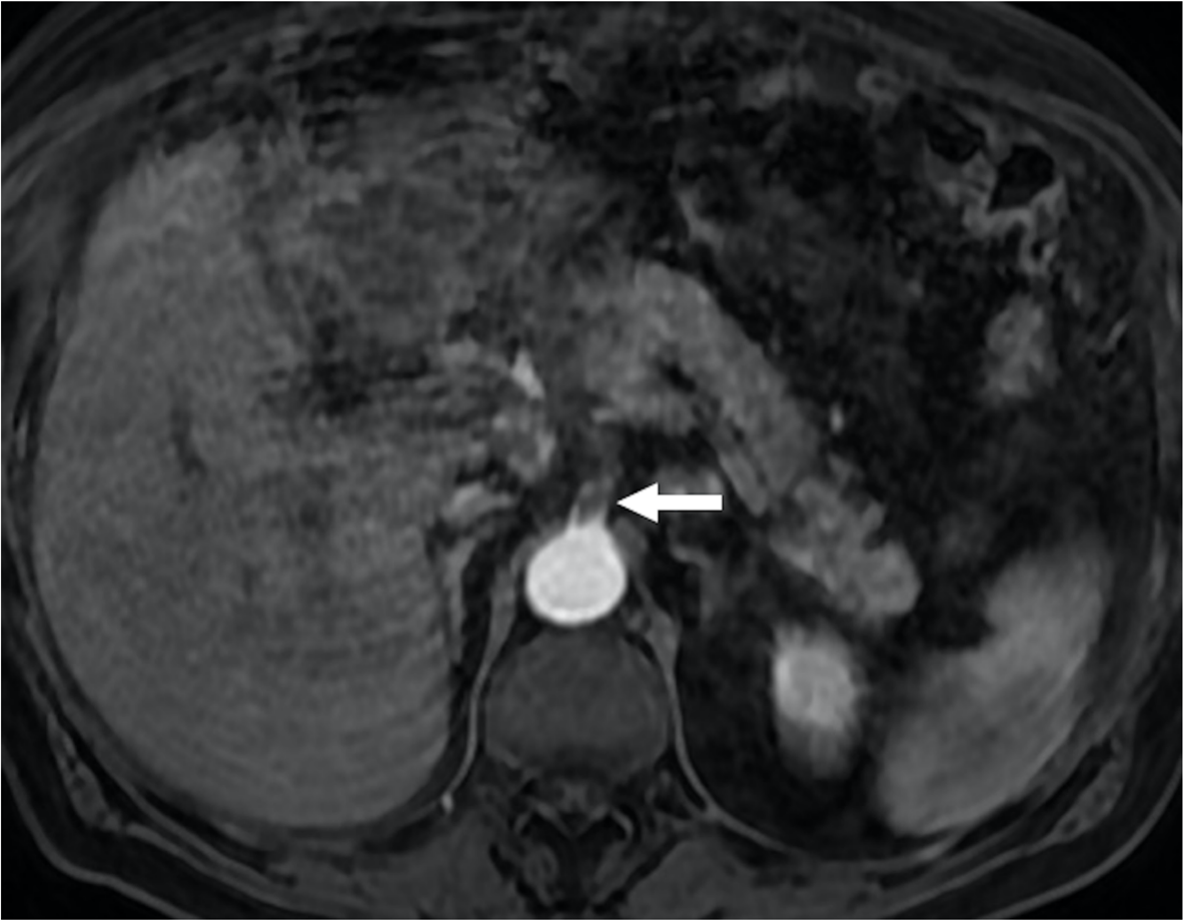
Celiac artery thrombosis. A 64-year-old male with known colon cancer treated with chemotherapy presented with severe epigastric pain and elevated liver enzymes. Axial plane post-contrast arterial phase T1W image shows intraluminal thrombus completely obstructing the celiac artery lumen (arrow). There was no extension of this thrombus into the aorta. Also, note was made of associated splenic infarcts (not shown)

It is frequently associated with other cardiovascular events. The most common etiology is atherosclerosis. Further, 20–30% of cases may have symptoms of chronic mesenteric ischemia [74]. Post-contrast CT and MRI examination may accurately detect acute celiac artery occlusion and associated end-organ ischemia (Fig. 27). The main goal of the treatment is to reestablish the diminished or stopped mesenteric blood flow and to avoid end-organ ischemia [75].

### Dunbar syndrome

Dunbar syndrome (the median arcuate ligament syndrome) is typically caused by compression of the celiac artery ostium by a low insertion of the median arcuate ligament. Abdominal pain may be observed in certain patients related to this phenomenon. CTA may outline the characteristic imaging feature of this syndrome. Post-stenotic dilatation and collateral vessels from SMA may be observed in patients with severe stenosis [76].

## Splenic artery

### Splenic artery aneurysm rupture

Splenic artery aneurysm (SAA) is a rare clinical condition with an estimated prevalence of 0.2–10.4% [77]. However, among visceral aneurysms, SAA is the most commonly detected aneurysm, representing around 60% of all splanchnic artery aneurysms [78]. The most important risk associated with SAAs is their tendency to rupture. The rupture is a true medical emergency and may cause life-threatening bleeding and is seen in 10% of the cases. The mortality is rare and may be extremely high during pregnancy (around 70%) [79]. Most patients with SAA are asymptomatic and diagnosis is typically incidental [80]. Treatment is typically indicated in aneurysm larger than 2 cm in diameter, in pregnant patients and patients undergoing major abdominal surgery [81].

**Fig. 28:**
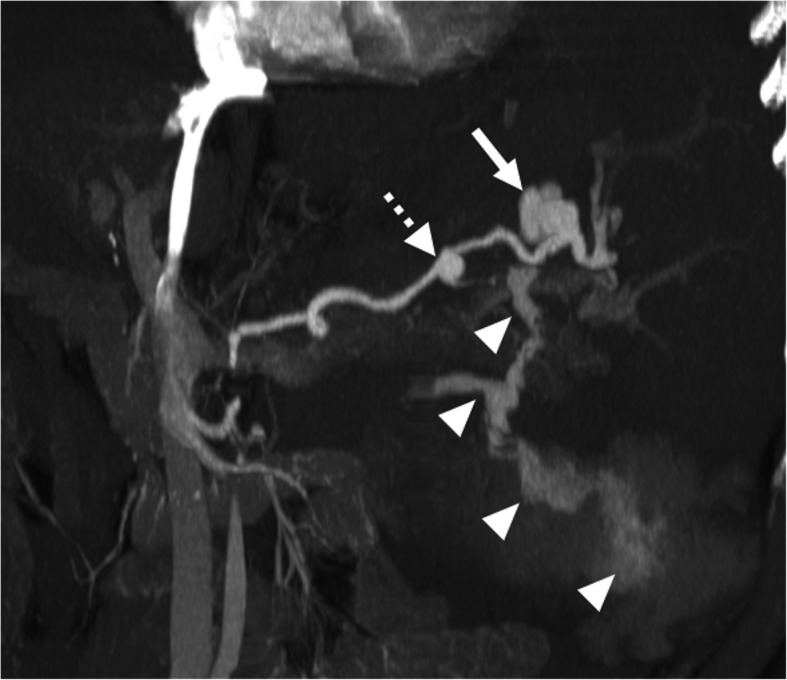
Splenic artery aneurysm. A 48-year-old male with known hepatitis B associated chronic liver disease presented to ED with acute onset severe abdominal pain and hypotension. Coronally reformatted post-contrast MIP image demonstrates a large lobulated splenic artery aneurysm (arrow) in the distal part of splenic artery. There was marked contrast extravasation from this aneurysm (arrowheads) consistent with acute rupture. Also, note is made of another smaller aneurysm (broken arrow) located in the proximal part of the splenic artery

CT is the most commonly used imaging modality for diagnosis. The SAAs typically reside in the distal third portion of the splenic artery (74–87%), followed by the middle third segment (22%). It is observed as an extra filling pouch along the course of the artery and may have peripheral wall calcifications, indicative of atherosclerotic changes (Fig. 28). Surgery, with excision, ligation, or revascularization with or without splenectomy, or endovascular approaches are the two main treatment options [81].

### Gastroduodenal artery pseudoaneurysm

Gastroduodenal artery pseudoaneurysms (GDAP) are most commonly seen after acute pancreatitis, or less commonly, after gastric or pancreatic surgeries [82]. Early diagnosis and treatment are imperative as rupture may cause life-threatening hemorrhage. Even small-sized GDAPs should also be treated when detected due to the high risk of rupture.

**Fig. 29:**
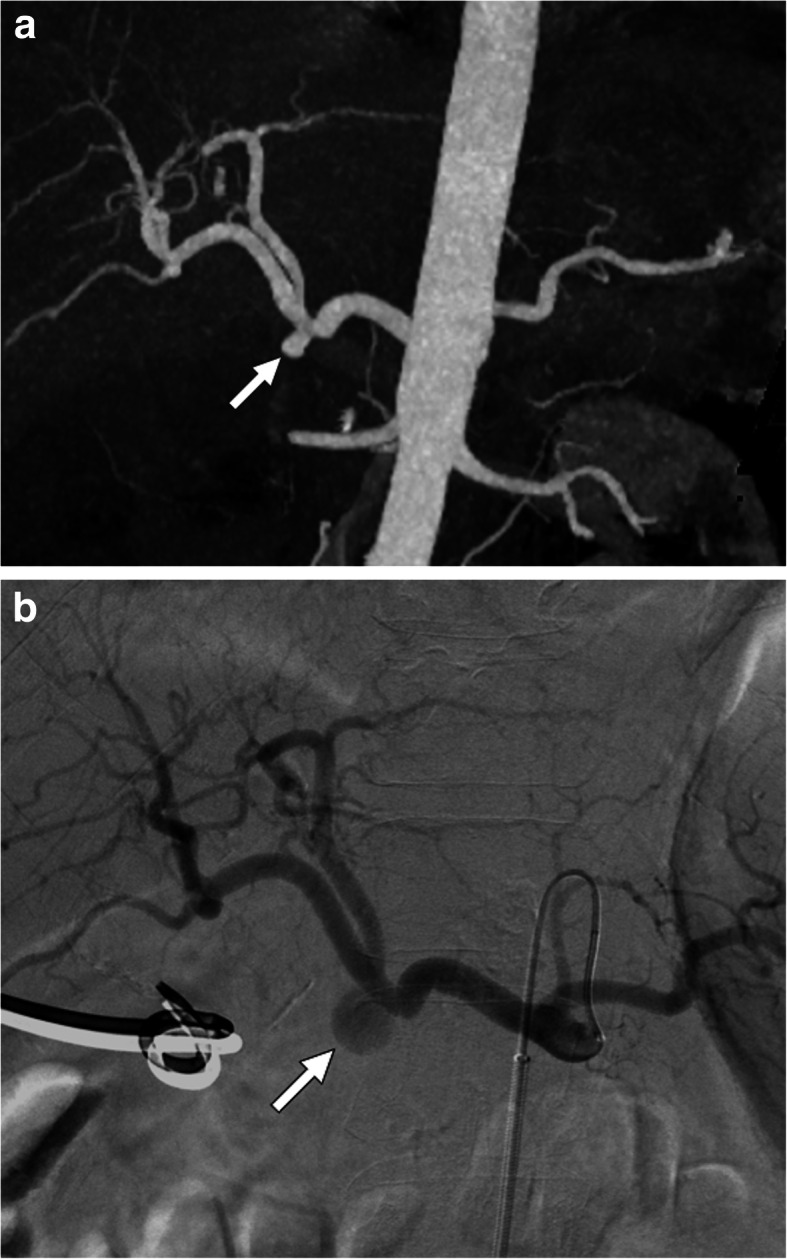
Gastroduodenal artery pseudoaneurysm. A 55-year-old male patient who underwent Whipple surgery for pancreatic head adenocarcinoma experienced severe abdominal pain and hypotension 2 days after the surgery. **a** MIP image of arterial phase abdominal CT angiography showed pseudoaneurysm (arrow) at the GDA stump. **b** Subsequent emergent catheter angiography confirmed the pseudoaneurysm (arrow) at the GDA stump. This pseudoaneurysm was successfully coil embolized

CT is the imaging modality of choice and allows detection in the majority of the cases (Fig. 29). Endovascular intervention, when feasible, is the most commonly used treatment approach.

## Conclusion

The diseases related to visceral arteries are not among the common causes of the acute abdomen but delay in diagnosis and treatment may carry a very high risk of morbidity and mortality. High index of clinical suspicion and meticulous evaluation of the imaging studies, as the findings may be extremely subtle, are two most important parameters for diagnosis and early intervention.

CTA should be the first-line modality to be utilized in these patients as it can provide an accurate diagnosis in a prompt manner. Appropriate protocoling of CTA is critical and a proper study should include non-contrast images followed by arterial and venous phases. Delayed phase images may also be implemented in clinically relevant situations under radiologists’ discretion. With CTA, both vascular structures and secondary effects of visceral vessel abnormalities can be simultaneously evaluated. Solid-organ infarcts, bowel wall abnormalities, the presence and extent of intraabdominal free fluid, and hematomas may also be precisely assessed with CT. The role of CT may be even more accentuated in patients who are hemodynamically unstable and need urgent treatment.

In addition to initial diagnosis, CT has also a significant potential to guide the endovascular, medical, or surgical treatment approaches as well as in follow-up of these patients.

## Data Availability

Data sharing is not applicable to this article as no datasets were generated or analyzed during the current study**.**

## Abbreviations

AAP: Acute abdominal pain
AMA: Aorto-mesenteric angle
AVF: Arteriovenous fistula
BCS: Budd-Chiari syndrome
CT: Computed tomography
ED: Emergency department
GDAP: Gastroduodenal artery pseudoaneurysm
HA: Hepatic artery
HAA: Hepatic artery aneurysm
HCC: Hepatocellular carcinoma
HV: Hepatic vein
LRV: Left renal vein
MRI: Magnetic resonance imaging
MVT: Mesenteric venous thrombosis
NCS: Nutcracker syndrome
OVT: Ovarian vein thrombosis
PV: Portal vein
PVT: Portal vein thrombosis
RAA: Renal artery aneurysm
RAP: Renal artery pseudoaneurysm
RVT: Renal vein thrombosis
SAA: Splenic artery aneurysm
SMA: Superior mesenteric artery
SMV: Superior mesenteric vein
SV: Splenic vein
US: Ultrasonography
